# Global, regional, and national burdens of eating disorders from 1990 to 2021 and projection to 2035

**DOI:** 10.3389/fnut.2025.1595390

**Published:** 2025-08-11

**Authors:** Xiangrong Liu, Lu Liu, Hang Yu, Xueyan Yang, Zhao Liu, Zhimeng Yu, Xin Qin, Yinghua Liu

**Affiliations:** ^1^Department of Nutrition, The First Medical Center of the Chinese People’s Liberation Army General Hospital, Beijing, China; ^2^People’s Liberation Army Postgraduate Medical School, Beijing, China

**Keywords:** eating disorder, anorexia nervosa, bulimia nervosa, incidence, mortality, disability-adjusted life years, global burden of disease

## Abstract

**Background:**

Eating disorders severely impact the physical and mental health and challenge global healthcare. This study examined global trends in incidence, mortality, and disability-adjusted life years (DALYs) related to eating disorders from 1990 to 2021 and projected the burden to 2035.

**Methods:**

Data from Global Burden of Disease (GBD) 2021 were used to calculate mortality, incidence, and DALYs rates. Analyses were stratified by age, sex, disorder type, and region. Frontier analysis quantified the gap between current and minimum achievable burdens. Decomposition analysis assessed population growth, aging, and epidemiological transitions. Health inequalities were studied using inequality indices. Future trends were predicted using Bayesian Age-Period-Cohort (BAPC) modeling.

**Results:**

The age-standardized DALYs rate for eating disorders increased from 37.33 (95% UI: 22.67–58.60) to 43.36 per 100,000 (95% UI: 26.35–68.45), and the age-standardized incidence rate (ASIR) rose from 106.78 (95% UI: 74.30–150.89) to 124.4 per 100,000 (95% UI: 86.48–175.74). The age-standardized DALYs rate (EAPC = 0.67) and ASIR (EAPC = 0.55) increased at faster annual rates for bulimia nervosa than for anorexia nervosa. In 2021, the highest age-standardized death rate (ASDR) were recorded in Central Europe, the largest age-standardized DALYs rate were documented in Western Europe, and the greatest ASIR were reported in Andean Latin America. High sociodemographic index (SDI) regions bore the greatest burden. We also found that Females, particularly aged 15–24, experienced higher burdens. Decomposition analysis underscored the variations in the drivers of disease burden across different SDI regions. Analysis of health inequality showed that the disparity in disease burden attributable to economic factors has further widened. BAPC modeling predicted continued burden growth.

**Conclusion:**

The disease burden imposed by eating disorders is gradually increasing, especially impacting women, youth, and young adults, and more so in regions with a high SDI index. Projections indicate that by 2035, this burden will still be substantial. Health inequalities due to the wealth gap have become more severe. These findings can guide targeted strategies for prevention and control.

**Plain English summary:**

This is the first study to use the GBD 2021 data to provide a comprehensive in-depth study of the global, regional, and national burdens of eating disorders between 1990 and 2021. In this study, we explored the deeper drivers of the disease burden using frontier, decomposition, and health inequality analyses, in addition to describing the burden of mortality, morbidity, and DALYs in eating disorders. Potential trends through 2035 were also projected using BAPC modeling. We found that the disease burden imposed by eating disorders was gradually increasing. Health inequalities due to the wealth gap had become more severe. This study offered the most current and comprehensive global assessment of the burden of eating disorders, providing critical and updated evidence for healthcare professionals, public health practitioners, and policymakers worldwide. Emphasizing the holistic treatment of mental and physical health, and fostering global collaboration and data sharing were critical for advancing the study and prevention of eating disorders.

## Introduction

Eating disorders are a complex set of psychiatric disorders characterized by abnormalities in eating behaviors, cognitive traits, and psychological symptoms (1). These disorders are usually characterized by an excessive preoccupation with food, distorted perceptions of weight and body size, and resulting emotional problems and social dysfunction. Eating disorders include the following types: anorexia nervosa, bulimia nervosa, binge eating disorder (BED), avoidant food intake disorder, and other specified feeding or eating disorders (OSFEDs). Anorexia nervosa is a disorder characterized by extreme dieting, underweight, and intense fear of weight gain. Patients may engage in extreme weight loss behaviors, such as self-induced vomiting and excessive exercise. By contrast, bulimia nervosa is characterized by recurrent episodes of overeating (ingesting large amounts of food in a short time) and subsequent inappropriate compensatory behaviors, such as self-induced vomiting, laxative abuse, strict dieting, or excessive exercise to prevent weight gain (1). In general, eating disorders have increased in prevalence over the last fifty years (2).

The onset of eating disorders may be related to a variety of factors, including genetic, environmental, and psychosocial elements, and their prevalence and manifestations may vary in different regions and cultures (3). In one study, the weighted prevalence of eating disorders was 5.2%, with anorexia nervosa accounting for 0.01%, bulimia nervosa for 0.6%, BED for 1.4%, and OSFEDs for 1.6% (4). Untreated eating disorders can have serious consequences, including death (5). Anorexia nervosa, in particular, has the highest mortality rate of any mental health disorder (6). One meta-analysis found a significant association between eating disorders and the risk of death, with a standardized mortality ratio (SMR) of 4.42 (95% CI: 3.55–5.50; *Z* = 13.31; *P* < 0.001), and an SMR of 5.31 for anorexia nervosa and the risk of death (7). Eating disorders also lead to impaired psychosocial functioning through self-image problems, social impairment, and occupational dysfunction (8). They also impose a significant economic burden on individuals, families, and society. Therefore, eating disorders are serious public health problems that require timely identification, diagnosis, and treatment to improve the prognosis of patients and to reduce the harm they cause to individuals and society (9).

Although eating disorders are attracting increasing attention, the latest in-depth epidemiologic evidence for them is still relatively sparse. The aim of the present study was to use the Global Burden and Disease Study (GBD) 2021 data to provide a comprehensive in-depth study of the global, regional, and national burdens of eating disorders between 1990 and 2021. While previous studies have utilized GBD 2017 data to reveal annual changes and trends in the prevalence of eating disorders and disability-adjusted life years (DALYs) at the global, regional, and national levels (10), the present study explored the deeper drivers of the disease burden using frontier, decomposition, and health inequality analyses, in addition to describing the burden of mortality, morbidity, and DALYs in eating disorders. Potential trends through 2035 were also projected using Bayesian age-part cohort (BAPC) modeling. The results of these analyses provide the most comprehensive epidemiological analysis of the burden of eating disorders to date and can serve as a reference for the selection of research directions by researchers and for policy formulation by relevant government officials.

## Materials and methods

All data in this study were obtained from the GBD 2021 dataset.^1^The GBD is a comprehensive database that records data on 371 diseases and injuries and 88 corresponding major risk factors in 204 countries and territories. Indicators in the database include incidence, prevalence, mortality, years of life lost, years lived with disability, and DALYs. The sociodemographic index (SDI) was also used to assess the overall development of a country or region, as it reflects a number of social factors, such as the level of economic development, educational attainment, and health conditions of a region (11). Specifically, the SDI contains three indicators: (1) the total fertility rate for individuals under the age of 25, (2) the average educational attainment for those aged 15 and above, and (3) the distribution of income per capita with temporal lag (12). The SDI value ranges from 0 to 1, with larger values representing higher levels of local economic and social development (13, 14). In this study, the SDI values were categorized into five levels: low-SDI [0–0.47), low-middle SDI [0.47–0.62), middle SDI [0.62–0.71), high-middle SDI [0.71–0.81), and high SDI [0.81–1.00]. The database collected data only for deaths related to anorexia nervosa; therefore, the death-related indicators for anorexia nervosa were used as the death-related indicators for eating disorders (12).

We used three key metrics to examine the burden of eating disorders by calculating the mortality rate, the incidence and DALYs rate, and the 95% uncertainty interval (UI) per 100,000 population. In this study, the estimated annual percent change (EAPC) in age-standardized rate (ASR) was used to assess the epidemiologic trends of eating disorders from 1990 to 2021 and to compare differences across genders, disease types, and regions. Changes in the burden of disease over time were further examined for five regions differing in SDI level. We also analyzed the correlation of ASR with EAPC and SDI and calculated Pearson correlation coefficients (15, 16).

We used an ASR-based frontier analysis to assess differences in the burden of disease between different countries and regions that differed in SDI level. This approach focused on determining the lowest theoretically achievable ASR value for each country or region based on its current SDI level, thereby quantifying the difference between the current burden of disease and its theoretically lowest possible burden. For countries that had not reached the frontier, the distance from the frontier was calculated to reveal potential room for improvement. We used a combination of locally estimated scatterplot smoothing and local polynomial regression techniques, using a smoothing parameter of 0.3, to construct a smoothed borderline. To ensure the reliability of the analysis results, we performed 1,000 instances of self-help sampling (13). Decomposition analysis was used to determine the impact of population growth, aging, and epidemiologic shifts on the burden of disease (17).

With this approach, the total disease burden could be decomposed into the contribution of individual factors, thus identifying the factors that contributed the most, as well as their direction. The slope index of inequality (SII) and concentration index of inequality (CII) were utilized to examine the differences in health inequality in the populations’ age-standardized death rate (ASDR), age-standardized DALYs rate, and age-standardized incidence rate (ASIR). The SII is a measure of the strength of the association between health outcomes and socioeconomic status and reflects the absolute difference between the highest and lowest socioeconomic classes. A negative value of the SII indicates that health issues are more prevalent among groups with lower socioeconomic status, while a positive value indicates that health issues are more common among groups with higher socioeconomic status. A larger absolute value of the SII indicates a more severe health inequality (18). By contrast, the CII is an indicator of the strength of the association between health outcomes in the distribution of socioeconomic status. The CII value ranges from –1 to 1, with negative values indicating that health outcomes are more concentrated in lower socioeconomic groups and positive values indicating that health outcomes are more concentrated in higher socioeconomic groups. A CII value close to zero indicates a more equitable distribution.

The BAPC model is able to differentiate the impacts of age, period, and cohort on health outcomes. The modeling results help to understand changes in health patterns over time for different generations, as well as the impact on health of public health events or policy changes during a specific period. The use of integrated nested Laplace approximations (INLA), a computationally efficient Bayesian inference technique, avoided the long simulation times required by traditional Markov chain Monte Carlo methods. By approximating the posterior distributions, INLA quickly provided accurate parameter estimates and predictions for large-scale datasets. The BAPC model was capable of generating specific prediction rates for different age groups, as well as calculating age-standardized prediction rates to compare the health status of populations in different age groups (19, 20). Data analysis and visualization were achieved using R software, version 4.4.1, and *P* < 0.05 was considered statistically different. Ethical approval was not required, as GBD is an open source database.

## Results

According to the GBD 2021 data, the ASDR for eating disorders decreased from 2.92e-3 per 100,000 (95% UI:2.47e-3 to 3.19e-3) in 1990 to 2.87e-3 per 100,000 (95% UI:2.42e-3 to 3.24e-3) in 2021. During the same period, the age-standardized DALYs rate increased from 37.33 per 100,000 (95% UI: 22.67–58.60) to 43.36 per 100,000 (95% UI: 26.35–68.45), and the ASIR rose from 106.78 per 100,000 (95% UI: 74.30–150.89) to 124.4 per 100,000 (95% UI: 86.48 to175.74). The annual percentage change indicated that the ASDR declined at a faster rate for males (EAPC = –1.33) than for females (EAPC = –0.25). Conversely, the increase in age-standardized DALYs rate (EAPC = 0.72) and ASIR (EAPC = 0.55) was more rapid for males than for females over the same timeframe. The age-standardized DALYs rate (EAPC = 0.67) and the ASIR (EAPC = 0.55) increased at a faster annual rate for bulimia than for anorexia nervosa over the study period.

**FIGURE 1:**
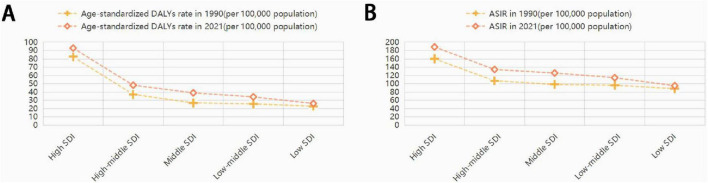
Age-standardized DALYs rate **(A)** and ASIR **(B)** of eating disorders in 5 SDI regions in 1990–2021. DALYs, disability-adjusted life years; ASIR, age-standardized incidence rate; SDI, socio-demographic index.

**FIGURE 2:**
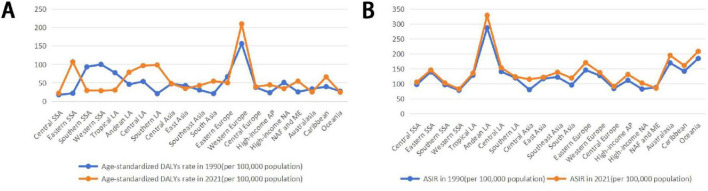
Age-standardized DALYs rate **(A)** and ASIR **(B)** of eating disorders in 21 Geographic Regions regions in 1990–2021. DALYs, disability-adjusted life years; ASIR, age-standardized incidence rate; SSA, Sub-Saharan Africa; LA, Latin America; AP, Asia Pacific; NA, North America; NAF, North Africa; ME, Middle East.

Between 1990 and 2021, the ASDR, age-standardized DALYs rate, and ASIR across different SDI quintiles showed an upward trend as the SDI index increased (Figure 1). Among the 21 regions analyzed, five regions experienced a decline in ASDR, and one region saw a decrease in age-standardized DALYs rate, whereas all regions observed an increase in ASIR. In 2021, Central Europe had the highest ASDR, Western Europe reported the greatest age-standardized DALYs rate, and Andean Latin America recorded the largest ASIR. The region with the largest average annual decrease in ASDR was southern Latin America (EAPC = –6.68), while Central Europe had the fastest increase (EAPC = 18.91). High-income North America showed a declining trend in age-standardized DALYs rate (EAPC = –0.17), whereas East Asia had the most rapid increases in age-standardized DALYs rate (EAPC = 1.79) and ASIR (EAPC = 1.17) (Table 1; Figure 2).

**TABLE 1:** ASDR, age-standardized DALYs rates and ASIR of eating disorders in 2021 and their temporal trend from 1990 to 2021 at global and regional levels.

	Death (95%UI)			DALYs(95%UI)			Incidence(95%UI)		
	ASR in 1990 (per 100,000 population)	ASR in 2021 (per 100,000 population)	EAPC (1990-2021)	ASR in 1990 (per 100,000 populatio)	ASR in 2021 (per 100,000 populatio)	EAPC (1990-2021)	ASR in 1990 (per 100 000 populatio)	ASR in 2021 (per 100,000 populatio)	EAPC (1990-2021)
Global	2.92e-3(2.47e-3, 3.19e-3)	2.87e-3(2.42e-3, 3.24e-3)	–0.32(–0.55, –0.10)	37.33(22.67, 58.60)	43.36(26.35, 68.45)	0.56(0.52, 0.61)	106.78(74.30, 150.89)	124.4(86.48, 175.74)	0.53(0.50, 0.56)
**Sex**									
Male	5.11e-4(1.44e-4, 6.22e-4)	3.73e-4(8.88e-5, 5.35e-4)	–1.33(–1.55, –1.11)	26.00(15.64, 41.22)	31.61(19.07, 50.47)	0.72(0.67, 0.78)	122.63(81.24, 178.91)	143.53(95.37, 209.56)	0.55(0.52, 0.59)
Female	5.39e-3(4.72e-3, 5.92e-3)	5.42e-3(4.67e-3, 6.07e-3)	–0.25(–0.49, –0.02)	48.93(29.97, 75.33)	55.48(33.98, 85.69)	0.47(0.44,0.51)	90.55(63.50, 122.12)	104.52(73.16, 142.06)	0.49(0.47, 0.52)
**Etiology**									
Anorexia nervosa	2.92e-3(2.47e-3, 3.19e-3)	2.87e-3(2.42e-3, 3.24e-3)	–0.32(–0.55, –0.10)	9.80(6.11, 15.53)	10.31(6.42, 16.25)	0.23(0.21,0.26)	14.84(10.34, 20.31)	16.59(11.66, 20.31)	0.42(0.39,0.45)
Bulimia nervosa				27.53(15.53, 45.89)	33.05(18.64, 55.29)	0.67(0.62,0.72)	91.95(57.24, 136.45)	107.80(66.79, 159.85)	0.55(0.52, 0.58)
**Socio-demogeaphic index**									
High SDI	1.23e-02(1.09e-02, 1.34e-02)	1.55e-02(1.37e-02, 1.70e-02)	0.358(0.018, 0.699)	82.33 (49.92, 127.21)	92.83 (57.08, 142.79)	0.32(0.28, 0.36)	159.98 (112.38, 220.09)	188.35 (131.96, 259.37)	0.47(0.45, 0.50)
High-middle SDI	2.72e-03(1.99e-03, 3.23e-03)	3.00e-03(2.34e-03, 3.52e-03)	0.419(–0.049, 0.890)	37.18 (22.80, 57.87)	48.19 (28.82, 76.48)	1.00(0.91, 1.10)	107.23 (74.56, 151.13)	134.46 (93.02, 189.67)	0.82(0.74, 0.89)
Middle SDI	3.75e-04(2.40e-04, 5.37e-04)	9.06e-04(6.00e-04, 1.33e-03)	2.607(2.002, 3.215)	26.80 (16.02, 42.47)	38.94 (23.55, 62.30)	1.32(1.28, 1.36)	98.14 (67.63, 140.41)	125.91 (86.51, 179.38)	0.83(0.80, 0.85)
Low-middle SDI	1.00e-04(5.70e-05, 1.41e-04)	2.43e-04(1.23e-04, 7.03e-04)	2.754(2.156, 3.356)	25.62 (15.60, 40.18)	34.14 (20.74, 54.35)	1.04(0.98, 1.10)	96.54 (66.55, 138.38)	114.58 (79.02, 163.21)	0.62(0.58, 0.65)
Low SDI	3.08e-06(5.57e-07, 1.46e-05)	3.65e-05(6.74e-06, 2.22e-04)	8.379(7.993, 8.766)	22.87 (13.88, 36.29)	26.13 (15.95, 41.83)	0.58(0.47, 0.69)	88.00 (60.84, 126.60)	95.15 (65.87, 136.44)	0.34(0.27, 0.40)
**Region**									
High-income Asia Pacific	1.53e-02(1.33e-02, 1.67e-02)	3.50e-04(1.51e-04, 6.46e-04)	0.67(–0.06, 1.40)	23.41 (14.22, 36.84)	44.69 (27.32, 69.41)	0.66(0.59, 0.73)	111.69 (78.50, 156.18)	131.14 (91.75, 183.40)	0.39(0.36, 0.42)
Tropical Latin America	1.83e-03(1.45e-03, 2.08e-03)	1.91e-02(1.68e-02, 2.14e-02)	0.08(–0.99, 1.16)	77.46 (47.74, 120.07)	30.59 (18.68, 49.24)	0.59(0.54, 0.64)	128.57 (89.27, 181.88)	135.66 (94.89, 191.48)	0.35(0.32, 0.37)
Central Asia	1.98e-03(8.86e-04, 3.32e-03)	4.35e-04(1.81e-04, 6.90e-04)	–1.29(–1.51, –1.07)	47.32 (28.39, 75.81)	48.11 (28.99, 76.64)	0.77(0.49, 1.05)	80.07 (54.31, 116.52)	114.96 (77.98, 166.76)	0.46(0.30, 0.63)
Western Sub-Saharan Africa	7.06e-06(1.95e-06, 1.01e-05)	1.98e-03(1.19e-03, 2.74e-03)	3.86(3.65, 4.08)	100.13 (60.19, 155.35)	28.74 (17.25, 45.58)	0.60(0.49, 0.72)	78.20 (53.95, 111.86)	82.35 (56.47, 118.20)	0.32(0.25, 0.39)
High-income North America	7.30e-03(6.27e-03, 7.95e-03)	8.53e-03(6.72e-03, 1.07e-02)	1.54(1.12, 1.96)	51.15 (30.27, 81.33)	34.33 (20.81, 54.78)	–0.17(–0.27, –0.07)	82.42 (56.75, 118.54)	102.42 (70.81, 146.81)	0.24(0.18, 0.30)
Central Sub-Saharan Africa	7.19e-06(1.99e-06, 1.11e-05)	2.85e-05(1.21e-05, 4.52e-05)	3.54(3.06, 4.03)	17.62 (10.49, 27.86)	22.08 (13.06, 35.20)	0.11(–0.09, 0.32)	97.31 (68.13, 135.38)	105.61 (73.62, 147.90)	0.01(–0.12, 0.13)
Australasia	1.20e-02(8.60e-03, 1.52e-02)	4.47e-03(3.73e-03, 5.40e-03)	–0.26(–0.71, 0.20)	33.59 (20.57, 52.28)	25.17 (15.33, 40.14)	1.12(1.00, 1.24)	169.75 (118.58, 234.55)	195.00 (138.08, 267.18)	0.40(0.32, 0.48)
East Asia	3.25e-04(1.06e-04, 5.91e-04)	2.04e-04(5.55e-05, 1.09e-03)	6.87(6.35, 7.39)	42.68 (26.21, 67.81)	34.62 (21.15, 55.09)	1.79(1.70, 1.88)	117.15 (79.87, 165.37)	121.51 (83.85, 170.92)	1.17(1.10, 1.24)
Southeast Asia	3.11e-04(1.43e-04, 5.36e-04)	1.72e-05(6.72e-06, 2.47e-05)	0.17(–0.64, 0.98)	30.60 (18.66, 47.57)	42.77 (26.01, 66.70)	1.04(0.98, 1.09)	122.08 (82.82, 171.14)	138.60 (94.49, 193.97)	0.65(0.61, 0.70)
Eastern Europe	2.10e-03(1.08e-03, 3.44e-03)	9.92e-04(8.01e-04, 1.21e-03)	2.35(1.73, 2.97)	66.96 (40.64, 104.64)	50.13 (30.19, 79.45)	0.57(0.33, 0.82)	145.90 (104.42, 193.24)	170.41 (120.79, 228.07)	0.33(0.19, 0.47)
Caribbean	3.32e-05(1.97e-05, 4.40e-05)	1.69e-03(1.28e-03, 2.22e-03)	–1.05(–2.10, 0.01)	39.72 (24.43, 61.96)	66.14 (37.72, 105.76)	0.29(0.24, 0.33)	141.85 (97.54, 192.80)	161.12 (111.41, 219.52)	0.17(0.15, 0.20)
Andean Latin America	1.91e-04(5.99e-05, 2.87e-04)	1.26e-02(1.06e-02, 1.46e-02)	3.44(2.51, 4.38)	46.01 (27.60, 73.08)	79.07 (47.21, 123.71)	0.77(0.73, 0.81)	287.34 (197.79, 393.31)	328.99 (233.82, 445.88)	0.46(0.44, 0.48)
Oceania	9.42e-06(2.03e-06, 1.23e-05)	1.57e-03(6.94e-04, 3.39e-03)	0.65(0.52, 0.78)	27.30 (16.58, 43.41)	24.63 (14.96, 38.76)	0.05(–0.01, 0.11)	184.71 (128.33, 255.30)	208.46 (141.66, 294.10)	0.08(0.03, 0.13)
Eastern Sub-Saharan Africa	5.47e-06(1.50e-06, 7.78e-06)	4.48e-05(2.52e-05, 6.13e-05)	3.64(3.55, 3.73)	21.80 (13.15, 34.82)	107.11 (66.61, 164.65)	0.60(0.50, 0.69)	139.84 (94.30, 197.57)	146.13 (99.65, 205.70)	0.33(0.27, 0.40)
Western Europe	2.07e-02(1.78e-02, 2.31e-02)	1.25e-05(3.20e-06, 2.06e-05)	–0.40(–0.58, –0.23)	156.13 (93.65, 244.59)	209.80 (135.00, 308.24)	0.49(0.44, 0.53)	127.50 (88.96, 176.99)	137.53 (96.01, 189.75)	0.54(0.48, 0.59)
North Africa and Middle East	1.74e-04(6.03e-05, 3.63e-04)	2.27e-05(7.86e-06, 3.94e-05)	3.22(2.84, 3.61)	25.51 (15.07, 40.12)	54.53 (33.17, 85.93)	0.76(0.69, 0.83)	87.72 (59.93, 124.79)	85.08 (58.30, 120.58)	0.38(0.33, 0.43)
Central Europe	5.13e-05(2.60e-05, 8.61e-05)	2.56e-02(2.18e-02, 2.99e-02)	18.91(15.37, 22.57)	37.80 (22.78, 60.45)	39.49 (24.44, 62.80)	1.17(1.08, 1.25)	83.86 (57.79, 120.10)	91.11 (62.92, 129.78)	0.63(0.59, 0.68)
Central Latin America	4.43e-04(3.54e-04, 5.08e-04)	1.30e-02(9.51e-03, 1.78e-02)	0.72(–0.92, 2.38)	53.71 (31.59, 85.28)	96.86 (59.65, 147.80)	0.25(0.22, 0.28)	140.71 (96.28, 200.59)	153.89 (104.76, 216.83)	0.17(0.14, 0.20)
South Asia	2.92e-05(7.23e-06, 1.67e-04)	2.01e-03(1.63e-03, 2.32e-03)	6.45(6.21, 6.69)	20.97 (12.51, 32.97)	54.50 (32.63, 86.84)	1.33(1.28, 1.39)	95.84 (65.26, 139.78)	119.00 (80.96, 171.74)	0.77(0.73, 0.81)
Southern Latin America	4.52e-03(2.83e-03, 6.78e-03)	2.24e-05(8.93e-06, 3.68e-05)	–6.68(–9.76, –3.49)	20.75 (12.62, 32.66)	98.62 (59.94, 152.43)	0.56(0.51, 0.61)	119.69 (81.81, 172.44)	123.34 (84.96, 177.26)	0.38(0.35, 0.40)
Southern Sub-Saharan Africa	2.56e-05(1.61e-05, 3.62e-05)	6.00e-04(3.82e-04, 8.47e-04)	2.15(1.86, 2.44)	93.42 (57.78, 141.65)	29.53 (17.91, 46.84)	0.26(0.23, 0.29)	96.50 (66.92, 137.43)	102.80 (70.85, 147.37)	0.19(0.16, 0.22)

UI, uncertainty intervals; DALYs, disability-adjusted life years; ASR, age-standardized rate; EAPC, estimated annual percentage change.

**FIGURE 3:**
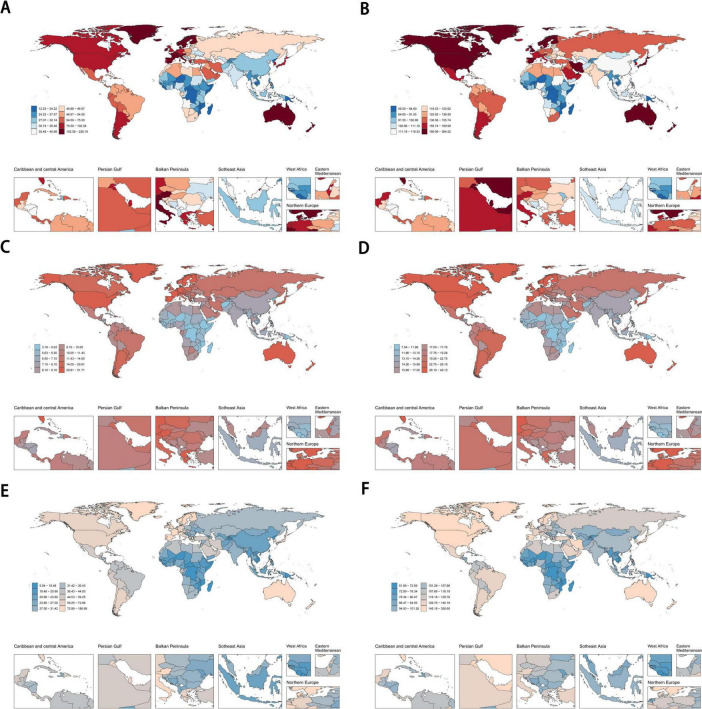
The age-standardized DALYs rate and the ASIR of eating disorders **(A,B)**, anorexia nervosa **(C,D)** and bulimia nervosa **(E,F)** across 204 countries and territories in 2021. DALYs, disability-adjusted life years; ASIR, age-standardized incidence rate.

At the country level, the top three countries with the highest ASDR for eating disorders in 2021 were Switzerland (0.06, 95% UI: 0.04–0.07, per 100,000), Japan (0.04, 95% UI: 0.03–0.05, per 100,000), and the Netherlands (0.04, 95% UI: 0.03–0.05, per 100,000) (Supplementary Figure S1). The countries with the highest age-standardized DALYs rate were Australia (225.90, 95% UI: 145.77–330.90, per 100,000), Monaco (193.35, 95% UI: 116.62–295.57, per 100,000), and New Zealand (132.69, 95% UI: 80.91–207.49, per 100,000) (Figure 3A). For the ASIR, Sweden had the highest rate at 380.71 (95% UI: 246.62–559.27, per 100,000), followed by Australia (338.06, 95% UI: 242.51–458.81, per 100,000) and New Zealand (286.76, 95% UI: 194.61–402.37, per 100,000) (Figure 3B). For anorexia nervosa specifically, the countries with the highest age-standardized DALYs rate were Monaco (51.20, 95% UI: 30.68–79.34, per 100,000), Australia (40.87, 95% UI: 24.75–64.91, per 100,000), and Luxembourg (40.79, 95% UI: 24.91–63.09, per 100,000) (Figure 3C). The highest ASIR for anorexia nervosa was observed in Australia (48.64, 95% UI: 34.34–69.67, per 100,000), followed by Spain (40.92, 95% UI: 28.45–57.53, per 100,000) and Monaco (39.76, 95% UI: 27.90–56.32, per 100,000) (Figure 3D). Regarding bulimia nervosa, the countries with the highest age-standardized DALYs rate were Australia (185.04, 95% UI: 116.93–281.98, per 100,000), Monaco (142.15, 95% UI: 82.83–230.51, per 100,000), and Italy (100.43, 95% UI: 60.30–159.70, per 100,000) (Figure 3E). The highest ASIR for bulimia nervosa was found in Sweden (347.13, 95% UI: 214.25–527.83, per 100,000), followed by Australia (289.42, 95% UI: 193.08–407.76, per 100,000) and New Zealand (247.54, 95% UI: 153.50–365.64, per 100,000) (Figure 3F; Supplementary Tables S1–S7).

**FIGURE 4:**
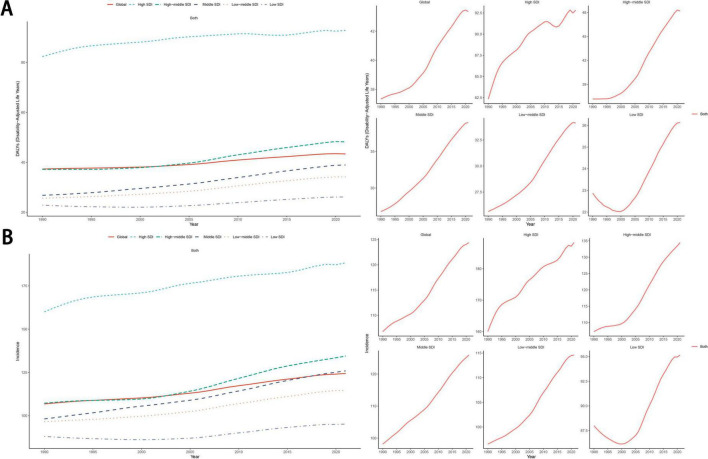
The trend of age-standardized DALYs rate **(A)** and ASIR **(B)** for eating disorders from 1990 to 2021. DALYs, disability-adjusted life years; ASIR, age-standardized incidence rate; SDI, socio-demographic index.

From 1990 to 2021, the age-standardized DALYs rates and the ASIR for eating disorders showed an increasing trend. Further analysis by five SDI quintiles revealed a significantly higher disease burden in the high SDI regions than in the other four SDI quintiles. Across all five SDI quintiles, the age-standardized DALYs rate and ASIR showed increasing trends over time. The same trend has also been observed in anorexia nervosa and bulimia nervosa (Figure 4; Supplementary Figure S2).

**FIGURE 5:**
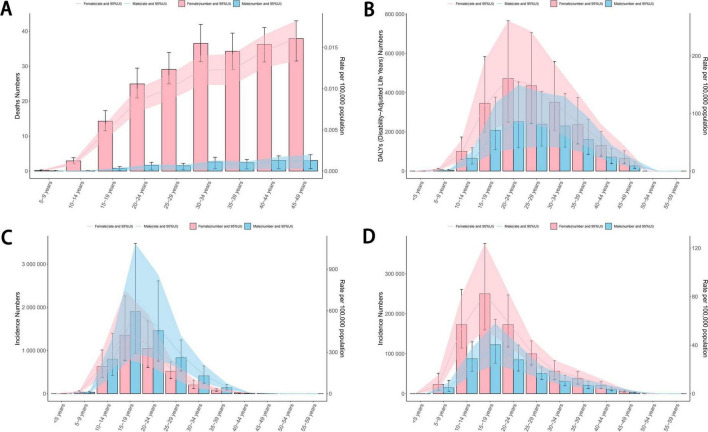
The ASDR **(A)**, age-standardized DALYs rate **(B)** and ASIR **(C)** per 100,000 people of eating disorders by age and sex in 2021. The ASIR **(D)** per 100,000 people of anorexia nervosa by age and sex in 2021. ASDR, age-standardized death rate; DALYs, disability-adjusted life years; ASIR, age-standardized incidence rate.

In 2021, for eating disorders, both the age-standardized DALYs cases and rate were higher in females than in males (Figure 5B). However, the age-standardized incidence cases and the ASIR showed an opposite trend, with higher values in males (Figure 5C). Both the age-standardized DALYs rate and ASIR for eating disorders showed a trend of initially increasing and then decreasing with age. The peak age group for the highest age-standardized DALYs rate was 20–24 years (Figure 5B), whereas the highest ASIR was observed at 15–19 years (Figure 5C). For anorexia nervosa, the ASDR correlated positively with age (Figure 5A). The trends for bulimia nervosa mirrored those of eating disorders as a whole, with the highest age-standardized DALYs rate and ASIR occurring in the same age groups. In the case of anorexia nervosa, the disease burden was greater in females than in males (Figure 5D; Supplementary Figure S3). Interestingly, the age groups with the highest age-standardized DALYs rate and incidence rate for anorexia nervosa were consistent with those for bulimia nervosa. However, there were certain differences in the age groups with the highest age-standardized DALYs rate among males (Supplementary Figure S3; Supplementary Table S8).

Based on the data from 1990, 2005, and 2021, the prevalence of bulimia nervosa was notably higher compared to anorexia nervosa, and no gender differences were apparent overall. Nevertheless, within the majority of Global Burden of Disease (GBD) regions, the proportion of bulimia nervosa was larger in males than in females (Supplementary Figure S4).

**FIGURE 6:**
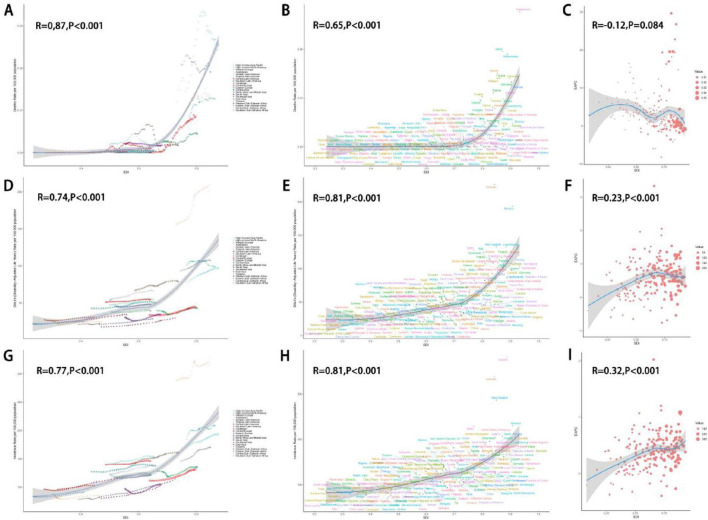
Association between ASR indicators of eating disorders and SDI. Eating disorders ASDR **(A–C)**, age-standardized DALYs rate **(D–F)**, ASIR **(G–I)**. ASR, age-standardized rate; SDI, socio-demographic index; EAPC, estimated annual percentage change. ASDR, age-standardized death rate; DALYs, disability-adjusted life years; ASIR, age-standardized incidence rate.

From 1990 to 2021, across the 21 GBD regions, the ASDR for eating disorders showed a positive correlation with the SDI (*R* = 0.87, *P* < 0.001) (Figure 6A), as did the age-standardized DALYs rate (R = 0.74, *P* < 0.001) (Figure 6D) and the ASIR (*R* = 0.77, *P* < 0.001) (Figure 6G). Similar positive correlations with SDI were observed for both anorexia nervosa and bulimia nervosa. In 2021, among the 204 countries worldwide, the ASDR, ASIR, and age-standardized DALYs rate for eating disorders also increased with higher SDI levels (Figure 6). Furthermore, the EAPC of age-standardized DALYs rate for eating disorders from 1990 to 2021 showed a significant positive correlation with the SDI level (*R* = 0.23, *P* < 0.001) (Figure 6F). The EAPC of the age-standardized DALYs rate for anorexia nervosa and bulimia nervosa increased with higher SDI levels (Supplementary Figure S5). Additionally, the EAPC of ASIR from 1990 to 2021 for eating disorders, anorexia nervosa, and bulimia nervosa increased with higher SDI levels, and this relationship was statistically significant (Supplementary Figure S5).

**FIGURE 7:**
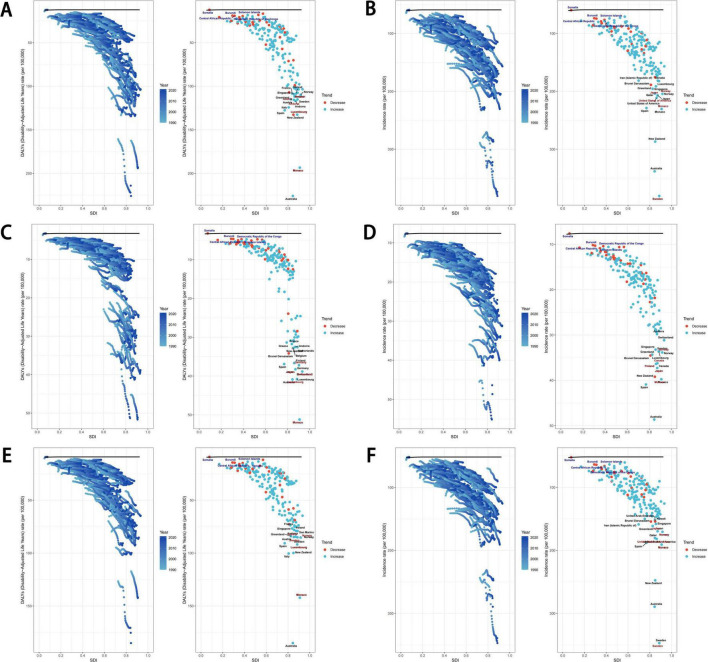
Frontier analysis exploring the relationship between SDI and ASR for eating disorders, anorexia nervosa and bulimia nervosa in 204 countries and territories. Age-standardized DALYs rate **(A)** and ASIR **(B)** of eating disorders, age-standardized DALYs rate **(C)** and ASIR **(D)** of anorexia nervosa, age-standardized DALYs rate **(E)** and ASIR **(F)** of bulimia nervosa. SDI, socio-demographic index; ASR, age-standardized rate; DALYs, disability-adjusted life years; ASIR, age-standardized incidence rate.

We conducted a frontier analysis to investigate the ideal scenario in which countries can control their disease burden relative to their SDI conditions. The five countries with the lowest SDI that were closest to the frontier fit line are marked in blue in Figure 5, while the five countries with the highest SDI that were farthest from the frontier fit line are marked in red. Additionally, the 15 countries that were farthest from the frontier fit line across all SDI levels are marked in black. Among low SDI countries, those closest to the frontier of the eating disorder burden were Somalia, Burundi, Solomon Islands, Central African Republic, and Democratic Republic of the Congo (Figure 7).

For higher SDI countries, the five nations farthest from the incidence frontier for both eating disorders and bulimia nervosa were Sweden, Monaco, the United States of America, Japan, and Norway (Figures 7B–F). The five countries farthest from the incidence frontier specifically for anorexia nervosa were Monaco, Japan, Finland, Canada, and Norway (Figure 7D). Additionally, the five countries farthest from the DALYs rate frontier for eating disorders were Monaco, Luxembourg, Andorra, Austria, and Sweden (Figure 7A).

**FIGURE 8:**
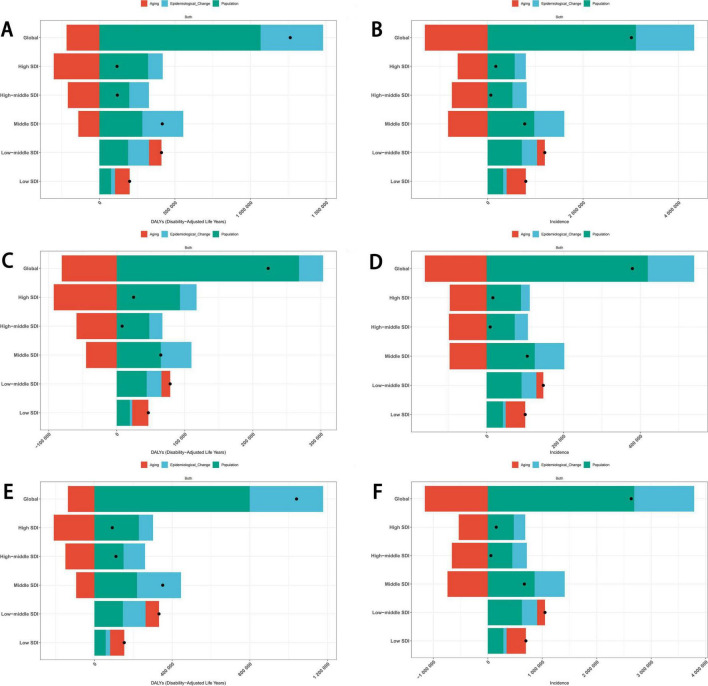
Decomposition analysis of the trends in eating disorders DALYs **(A)** and incidence **(B)**, anorexia nervosa DALYs **(C)** and incidence **(D)** and bulimia nervosa DALYs **(E)** and incidence **(F)** from 1990 to 2021. DALYs, disability-adjusted life years.

Overall, population growth and aging emerged as primary factors influencing the burden of eating disorders. Globally, population growth and epidemiological changes have exacerbated the DALYs and incidence burden of eating disorders. However, aging has had a mitigating effect on this burden in high SDI, high-middle SDI, and middle SDI regions. By contrast, in low-middle SDI and low SDI regions, aging has contributed to an increase in the DALYs and incidence burden of eating disorders (Figure 8). Similar trends were observed for anorexia nervosa and bulimia nervosa, with the same results as for eating disorders overall (Figure 8). In high SDI and high-middle SDI regions, epidemiological changes and population growth have positively influenced the mortality burden of eating disorders, while aging has had a negative influence. However, in middle SDI, low-middle SDI, and low SDI regions, all three factors—epidemiological changes, population growth, and aging—have positively contributed to the mortality burden of eating disorders (Supplementary Figure S6).

**FIGURE 9:**
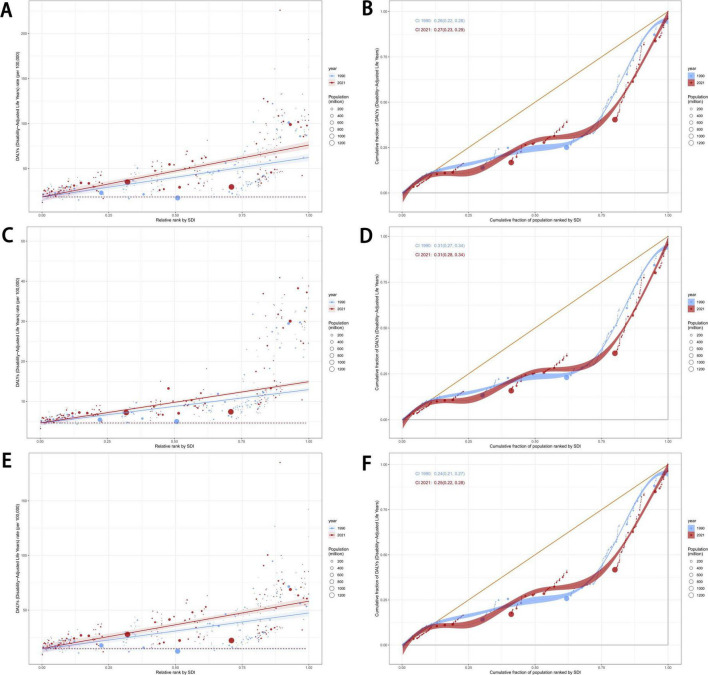
Health inequality regression curves and concentration curves for the age-standardized DALYs rate of eating disorder **(A,B)**, anorexia nervosa **(C,D)**, and bulimia nervosa **(E,F)**, 1990 and 2021. DALYs, disability-adjusted life years.

We also conducted an analysis of global health inequality. The SII of eating disorder age-standardized DALYs rate increased from 44.01 (95% CI: 36.11–51.90) in 1990 to 57.66 (95% CI: 49.00–66.32) in 2021, while the CII increased slightly from 0.26 (95% CI: 0.22–0.28) to 0.27 (95% CI: 0.23–0.29) in 2021. The SII of the anorexia nervosa age-standardized DALYs rate increased slightly, from 8.36 (95% CI: 7.19–9.54) in 1990 to 10.29 (95% CI: 9.11–11.47) in 2021, whereas the CII was unchanged. The SII of bulimia nervosa age-standardized DALYs rate increased from 33.06 (95% CI: 27.00–39.12) in 1990 to 43.21 (95% CI: 36.48–49.94) in 2021, while the CII increased slightly from 0.24 (95% CI: 0.21–0.27) to 0.25 (95% CI: 0.22–0.28) in 2021 (Figure 9).

The SII of eating disorders ASIR increased from 56.94 (95% CI: 47.37–66.50) in 1990 to 75.03 (95% CI: 65.68–84.37) in 2021, whereas the CII increased slightly from 0.12 (95% CI: 0.10–0.14) to 0.14 (95% CI: 0.12–0.16) in 2021. The SII of anorexia nervosa ASIR increased slightly from 10.61 (95% CI: 9.15–12.07) in 1990 to 12.70 (95% CI: 11.28–14.13) in 2021, while the CII increased slightly from 0.16 (95% CI: 0.14–0.18) to 0.17 (95% CI: 0.15–0.19) in 2021. The SII of bulimia nervosa ASIR increased from 45.47 (95% CI: 37.33–53.61) in 1990 to 61.16 (95% CI: 52.91 to 69.40) in 2021, while the CII increased slightly from 0.11 (95% CI: 0.09–0.13) to 0.13 (95% CI: 0.11–0.16) in 2021 (Supplementary Figure S7).

**FIGURE 10:**
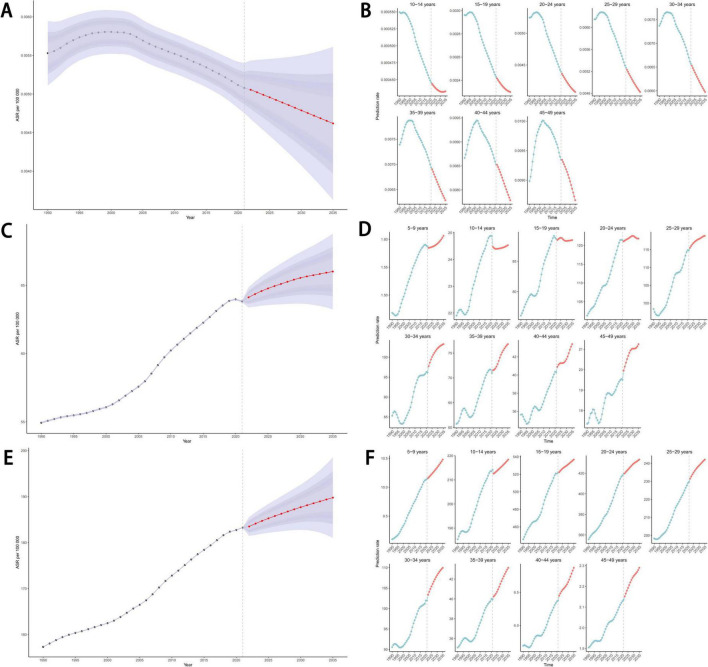
Projections of eating disorders ASDR **(A,B)**, age-standardized DALYs rate **(C,D)** and ASIR **(E,F)** by 2035 based on the BAPC model. ASDR, age-standardized death rate; DALYs, disability-adjusted life years; ASIR, age-standardized incidence rate; BAPC, Bayesian Age-Period-Cohort.

We used the BAPC model to forecast the disease burden of eating disorders, anorexia nervosa, and bulimia nervosa up to 2035. Globally, the mortality rate for eating disorders was projected to decline, while the age-standardized DALYs rate and ASIR were expected to increase; this trend was observed across all age groups (Figure 10). Conversely, for both bulimia nervosa and anorexia nervosa, the age-standardized DALYs rate and ASIR were predicted to rise, with this trend also being consistent across all age groups (Supplementary Figure S8).

## Discussion

Based on the latest update of the GBD 2021, we examined death, DALYs, and occurrences of eating disorders, anorexia nervosa, and bulimia nervosa at the global, regional, and national levels from 1990 to 2021, and we projected epidemiologic trends through 2035. Globally, ASDR showed a decreasing trend, while age-standardized DALYs rate and ASIR showed increasing trends for eating disorders and their subtypes. In addition, the burden of disease for eating disorders was significantly correlated with the SDI level. The study findings also confirmed that frontier analyses can help identify countries with the most urgent need to focus on combating eating disorders. Decomposition analyses found that population growth and epidemiologic changes had positive influences on the increasing burden of eating disorders, whereas the effect of aging was negative and varied considerably across SDI index regions. Our analysis of health inequality regarding age-standardized DALYs rate and ASIR for eating disorders revealed that the overall impact of the wealth gap is worsening. The BAPC model predicted a downward trend in ASDR for eating disorders, anorexia nervosa, and bulimia nervosa by 2035, while both the age-standardized DALYs rate and the ASIR are both trending upward.

The study found that the regions with the highest burden of disease for eating disorders included Western Europe, Australia, and New Zealand, and the region with the greatest increase in age-standardized DALYs rate and ASIR was East Asia. The ASIR for eating disorders, anorexia nervosa, and bulimia nervosa was greater for males than for females. Eating disorders, anorexia nervosa, and bulimia nervosa all had the highest age-standardized DALYs rate at 20–24 years of age and the highest ASIR at 15–19 years of age. The eating disorder disease burden increased with increasing levels of SDI, and EAPC was similarly positively correlated with SDI levels. These findings are in general agreement with a previous study by Wu et al. (10). Most of the countries in East Asia are developing countries, and are therefore more resource constrained. This hampers the prevention, detection, and treatment of these diseases, especially through the inadequate training of health professionals (21). A more important reason may be the changing food environment, as these countries may be facing more rapid social and environmental changes that could exacerbate the increase in eating disorders (4).

Although the EAPC of the age-standardized DALYs rate and the ASIR for eating disorders were significantly and positively correlated with the level of SDI, the EAPC increased and then decreased with SDI, which partly explains why the EAPC of disease burden was greatest in East Asia. An Australian study (22) found that DALYs for eating disorders were higher in females, with eating disorders being twice as prevalent in females than in males. Data from Chile and Spain also show that the prevalence of anorexia nervosa is much higher in women than in men (23, 24). Similar findings have been reported in other studies, with eating disorders predominantly affecting young women, especially adolescents and young adults (25). In many cultures, body image expectations for women tend to be more stringent, emphasizing “thinness” as beauty, and this sociocultural pressure may contribute to women’s greater vulnerability to developing eating disorders and an increased risk of anorexia nervosa (26). In addition, because anorexia nervosa is more common in women, healthcare professionals may be more likely to recognize and report cases in female patients, while ignoring or underdiagnosing male patients, and this may also partially explain the greater burden of anorexia nervosa observed in women (27).

In terms of age differences, young people are at a critical period of psychological and social development and are susceptible to socio-cultural norms and aesthetics, especially the pursuit of “thinness,” which may exacerbate the onset and development of eating disorders (28). Eating disorders are more prevalent in areas with high SDI, which may be related to higher social stress and aesthetic values in these areas (24, 29). Furthermore, populations in high SDI areas may have easier access to healthcare services, which may increase the rate of diagnosis and reporting of eating disorders (29).

The frontier analysis indicated that low SDI areas closest to the frontier could consider reducing their inputs in balancing dietary risks. The most striking example is that Somalia, Burundi, Solomon Islands, and the Central African Republic already have good control of the burden of eating disorders, including anorexia nervosa and bulimia nervosa. By contrast, areas with higher SDI areas should consider increasing their health economic inputs to attenuate the risk of eating disorders. In fact, further emphasis should be placed on the tumor burden attributable to diet in all regions far from the frontier. In particular, Sweden, Monaco, Japan, and Norway are regions with high levels of economic development, but the tumor burden attributable to diet remains significant in those countries and requires more urgent attention and resolution.

Decomposition analysis revealed that the burden of eating disorders, anorexia nervosa, and bulimia nervosa is increasing due to the impact of population growth and epidemiologic changes. As the population grows, competitive pressures and the pace of life increase, family structures and social support systems may change, and these pressures and changes may affect an individual’s mental health and coping mechanisms, which may exacerbate the onset and progression of eating disorders (30). Population growth may lead to an unequal distribution of healthcare resources and services, especially in rural and remote areas. This inequality can possibly lead to difficulties in accessing timely and effective treatment for people with eating disorders, thus affecting their recovery and quality of life (31). Population growth is also often accompanied by cultural exchange and integration, and the spread of the pursuit of thinness common in Western cultures through population growth and cultural exchange may explain the increased prevalence of eating disorders in non-Western countries (3).

The identification and diagnosis of eating disorders may become more effective as diagnostic technology improves, as advances in blood biomarkers and neuroimaging technology continue to improve the understanding of the biological basis of these disorders (32). Eating disorders may be comorbid with other psychiatric disorders, such as anxiety disorders, obsessive-compulsive disorders, and depressive disorders (33), while patients with severe mental disorders requiring long-term care and treatment have a higher prevalence of physical illness and premature death (34). Together, these factors contribute to the epidemiologic changes that can worsen the burden of eating disorders, anorexia nervosa, and bulimia nervosa. Aging exacerbates the burden of eating disorders in low-SDI and low-middle SDI areas. The opposite effect observed in higher SDI areas may reflect the fact that higher SDI areas typically have more developed economies and better healthcare systems, which leads to higher awareness and prevention of eating disorders among residents. In addition, residents of higher SDI areas may be less exposed to major risk factors associated with eating disorders (35).

Health inequality analysis showed a slight decrease in age-standardized DALYs rate SII and a more pronounced decrease in CII for eating disorders, anorexia nervosa, and bulimia nervosa between 1990 and 2021. This suggests that a decrease has occurred in the inequality in tumor burden between poor and rich countries, and that the gap between rich and poor may be narrowing in some regions. The BAPC prediction model shows that the burden of death in eating disorders is decreasing, but the age-standardized DALYs rate and ASIR are continuing to increase. In fact, the ASDR for eating disorders is undergoing a process of first increasing and then decreasing over time. The decrease in ASDR may be attributed to the growing public awareness of eating disorders, which facilitates early diagnosis and intervention, with improvements in treatments also acting as a major factor (36). Nevertheless, the current burden of disease due to eating disorders remains severe, and a combination of policy support, positive media culture, and economic and medical research will help reduce the impact of eating disorders on individuals and society.

Two notable findings were that the ASDR for anorexia nervosa trended downward and ASIR trended upward between 2018 and 2021. Age-standardized DALYs rate trended downward between 2019 and 2021; however, a retrospective study in New Zealand showed an increase in the prevalence and hospitalization of anorexia nervosa among females aged 10–19 years during the COVID-19 pandemic, with a particularly significant increase in the group aged 10–14 years (36). Globally, the prevalence of anxiety and depression also spiked by 25% in the first year of the corona virus epidemic (37). This may be related to the psychological stress and lifestyle changes that people faced during the pandemic, as these could exacerbate the symptoms of eating disorders. In addition, the COVID-19 pandemic added an additional challenge for people with eating disorders who were already vulnerable due to low body weight and reduced immunity (38). The reason for the accelerated rate of decline in ASDR for anorexia nervosa and the shift in age-standardized DALYs rate from an increasing to a decreasing trend from 2019 may also reflect the major disruptions caused by the pandemic in global health services, as reflected by delays and even interruptions in cancer diagnosis (39). These disruptions could have resulted in a decrease in the number of reports of eating disorders.

This study had the following limitations: (1) Similar to GBD 2017 (10), GBD 2021 failed to include BED and OSFEDs among eating disorders, and data on the burden of death in bulimia nervosa were not included, leading to a certain lack of comprehensiveness in the study. (2) The quality and level of exhaustiveness of the raw data also varies considerably across countries and regions. Certain low- and middle-income countries may lack high-quality surveillance systems, resulting in incomplete or unreliable data. Even in some high-income countries, health data for certain specific populations (e.g., migrants, homeless people, and residents of remote areas) may be underestimated or overlooked. Therefore, the compiled results may be somewhat biased.

## Conclusion

Between 1990 and 2021, the age-standardized DALYs rate and ASIR for eating disorders showed an increasing trend, while ASDR showed an increasing and then a decreasing trend. The burden of disease remains high, especially in females, youths, and young adults and in high SDI areas. Projections for 2035 remain bleak. Health inequalities, exacerbated by the wealth gap, have intensified. The results of the frontier analysis presented here will help governments and health administrations understand the current status of eating disorder prevention and treatment in their countries and adjust their health policies. Overall, the findings of this study provide new insights and reliable references for further global epidemiological studies on eating disorders.

## Data Availability

Publicly available datasets were analyzed in this study. This data can be found at: https://www.healthdata.org/gbd?spm=5176.28103460.0.0.6f0b451efkpcol.
