# Supplementary material for: Global, regional, and national burdens of eating disorders from 1990 to 2021 and projection to 2035
Source: Front Nutr. 2025 Aug 11;12:1595390. doi: 10.3389/fnut.2025.1595390 (PMC12375436; doi:10.3389/fnut.2025.1595390)
Supplement: Supplementary file 1 [file Image_1.pdf]

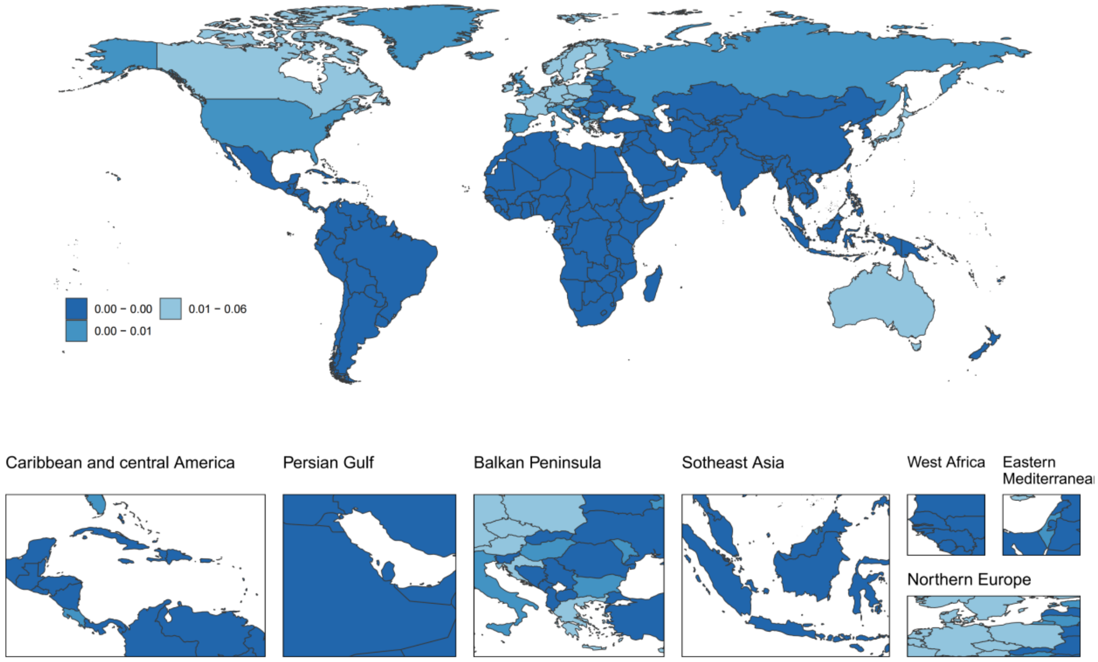

Fig. S1: The age-standardized ASDR of eating disorders across 204 countries and territories in 2021. ASDR, age-standardized death rate.

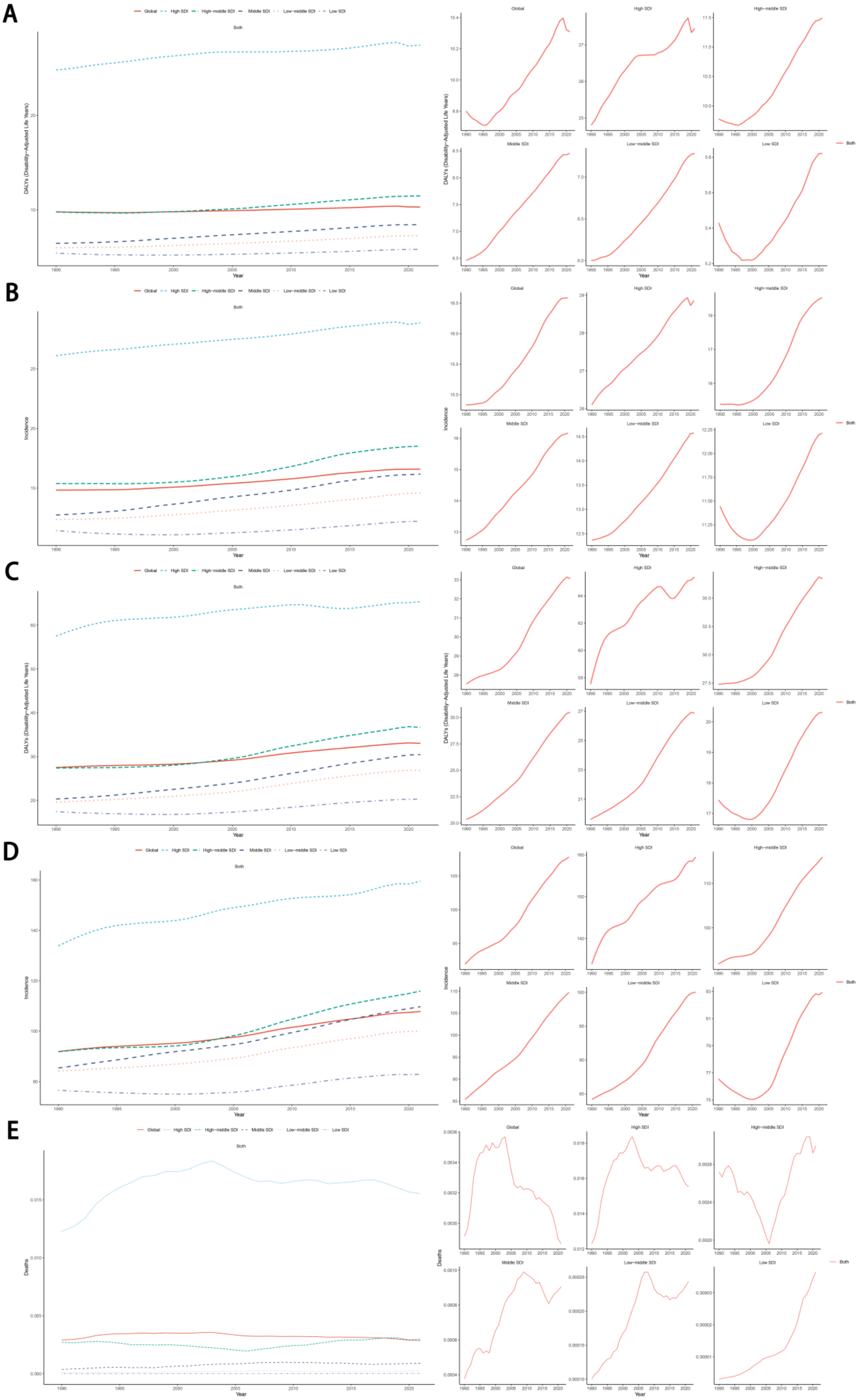
A
B
C
D
E
Global
High SDI
High-middle SDI
Middle SDI
Low-middle SDI
Low SDI
Both
DALYs (Disability-Adjusted Life Years)
Incidence
Deaths
Year

Fig. S2: The trend of age-standardized DALYs rate and ASIR for anorexia nervosa (A and B) and bulimia nervosa (C and D) from 1990 to 2021. The trend of ASDR for anorexia nervosa (E) from 1990 to 2021. DALYs, disability-adjusted life years; ASIR, age-standardized incidence rate; ASDR, age-standardized death rate; SDI, socio-demographic index.

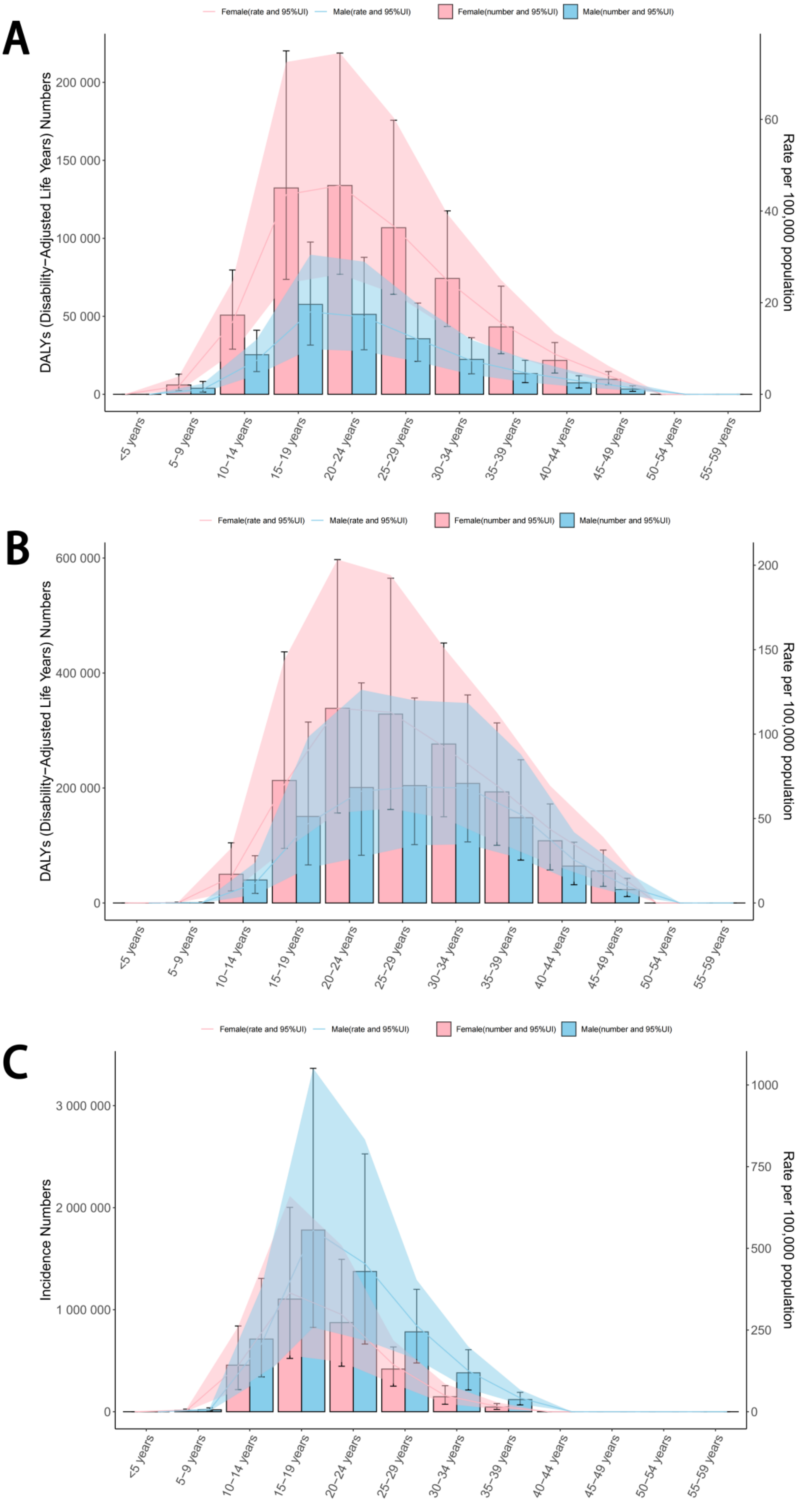

A
Female(rate and 95%UI)
Male(rate and 95%UI)
Female(number and 95%UI)
Male(number and 95%UI)
DALYs (Disability−Adjusted Life Years) Numbers
0
50 000
100 000
150 000
200 000
Rate per 100,000 population
0
20
40
60
<5 years
5−9 years
10−14 years
15−19 years
20−24 years
25−29 years
30−34 years
35−39 years
40−44 years
45−49 years
50−54 years
55−59 years
B
Female(rate and 95%UI)
Male(rate and 95%UI)
Female(number and 95%UI)
Male(number and 95%UI)
DALYs (Disability−Adjusted Life Years) Numbers
0
200 000
400 000
600 000
Rate per 100,000 population
0
50
100
150
200
<5 years
5−9 years
10−14 years
15−19 years
20−24 years
25−29 years
30−34 years
35−39 years
40−44 years
45−49 years
50−54 years
55−59 years
C
Female(rate and 95%UI)
Male(rate and 95%UI)
Female(number and 95%UI)
Male(number and 95%UI)
Incidence Numbers
0
1 000 000
2 000 000
3 000 000
Rate per 100,000 population
0
250
500
750
1000
<5 years
5−9 years
10−14 years
15−19 years
20−24 years
25−29 years
30−34 years
35−39 years
40−44 years
45−49 years
50−54 years
55−59 years

Fig. S3 : The age-standardized DALYs rate (A) per 100 000 people of anorexia nervosa by age and sex in 2021.The age-standardized DALYs rate (B) and ASIR (C) per 100 000 people of bulimia nervosa by age and sex in 2021. DALYs, disability-adjusted life years; ASIR, age-standardized incidence rate.

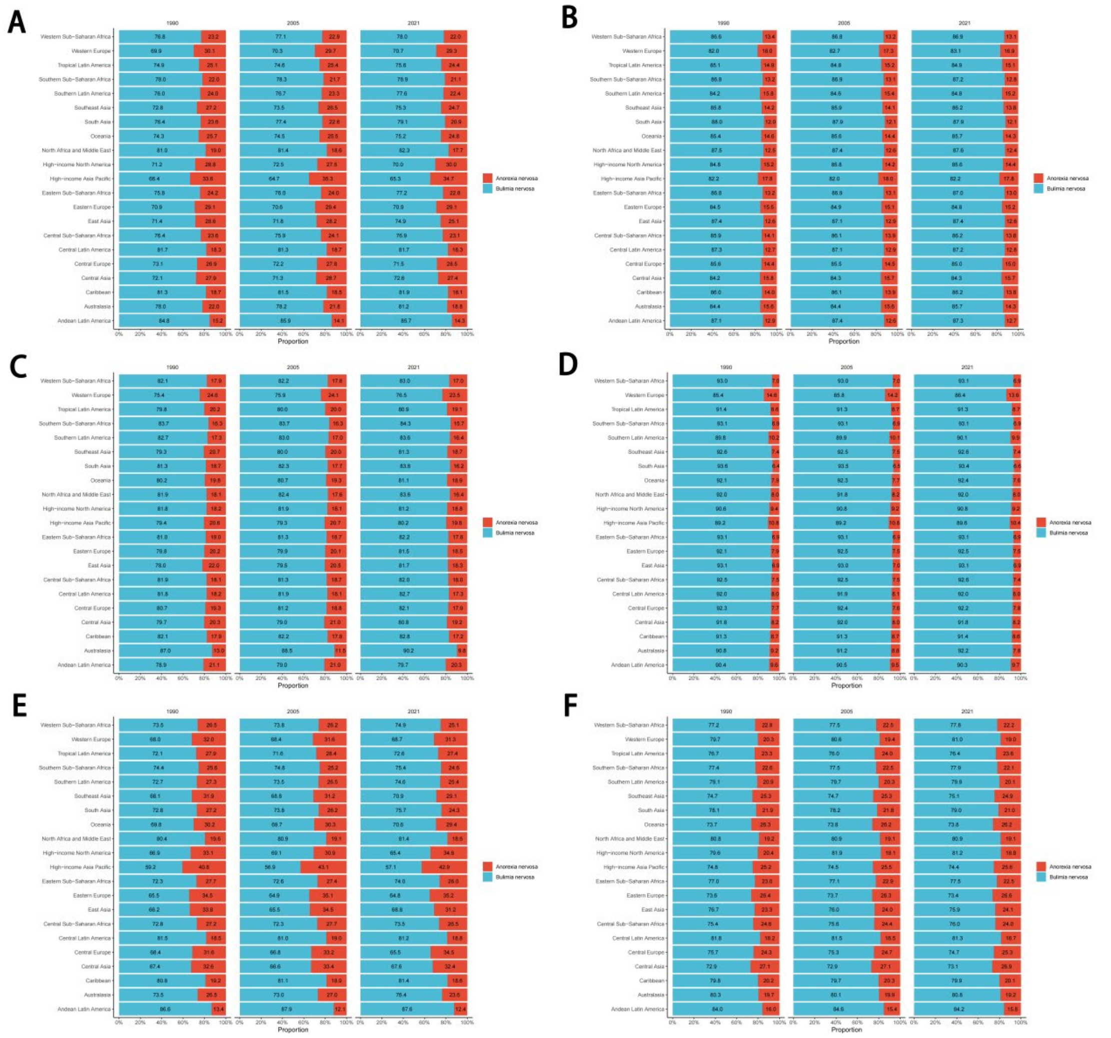

Fig. S4: The comparison of DALYs (A) and incidence (B) burden of anorexia nervosa and bulimia nervosa in the general population, and their situation in males (C-D) and females (E-F). DALYs, disability-adjusted life years.

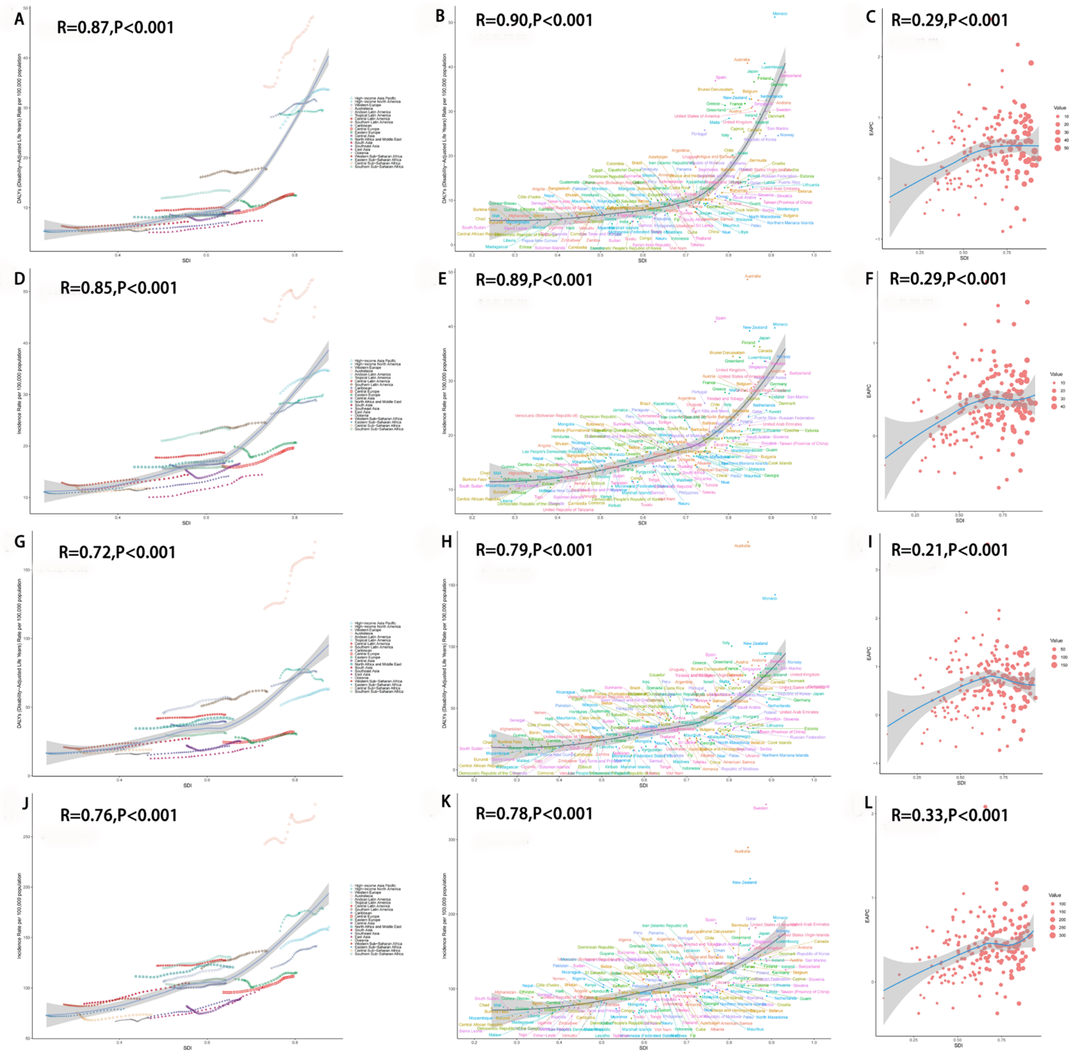

Fig. S5 : Association between ASR indicators of eating disorders and SDI. Anorexia nervosa age-standardized DALYs rate (A-C), ASIR(D-F). Bulimia nervosa age-standardized DALYs rate (G-I), ASIR(J-L). ASR, age-standardized rate; SDI, socio-demographic index; EAPC, estimated annual percentage change. ASDR, age-standardized death rate; DALYs, disability-adjusted life years; ASIR, age-standardized incidence rate.

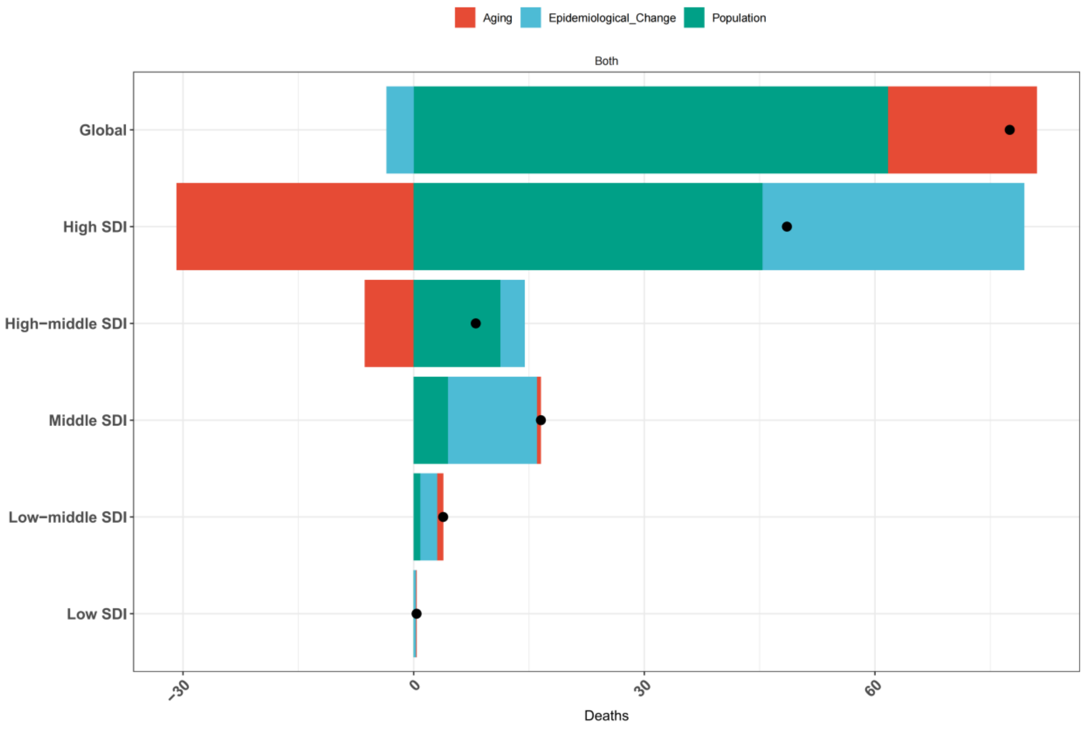

Fig. S6: Decomposition analysis of the trends in eating disorders death from 1990 to 2021.

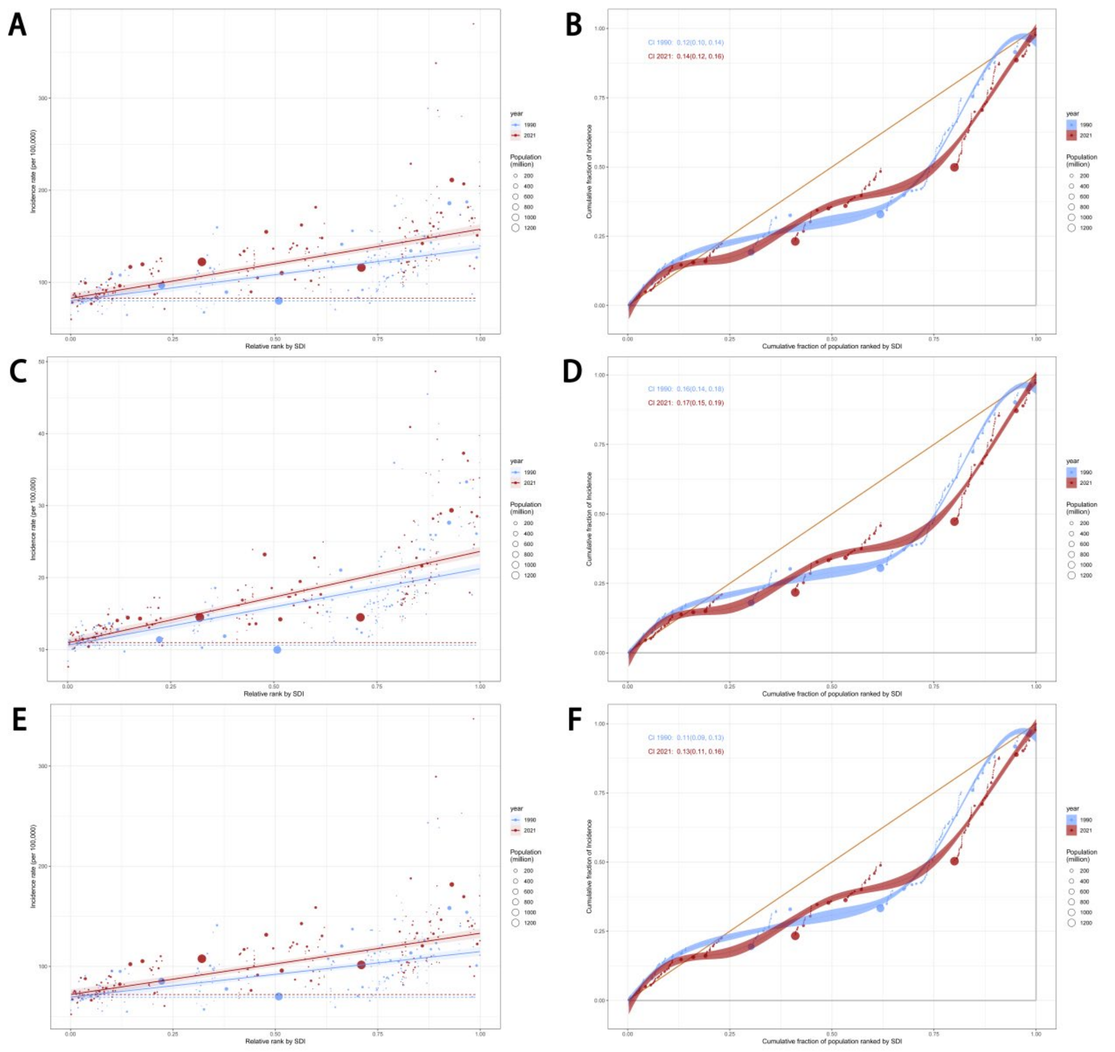

Fig. S7:Health inequality regression curves and concentration curves for the ASIR of eating disorder (A and B), anorexia nervosa (C and D), and bulimia nervosa (E and F), 1990 and 2021. ASIR, age-standardized incidence rate.

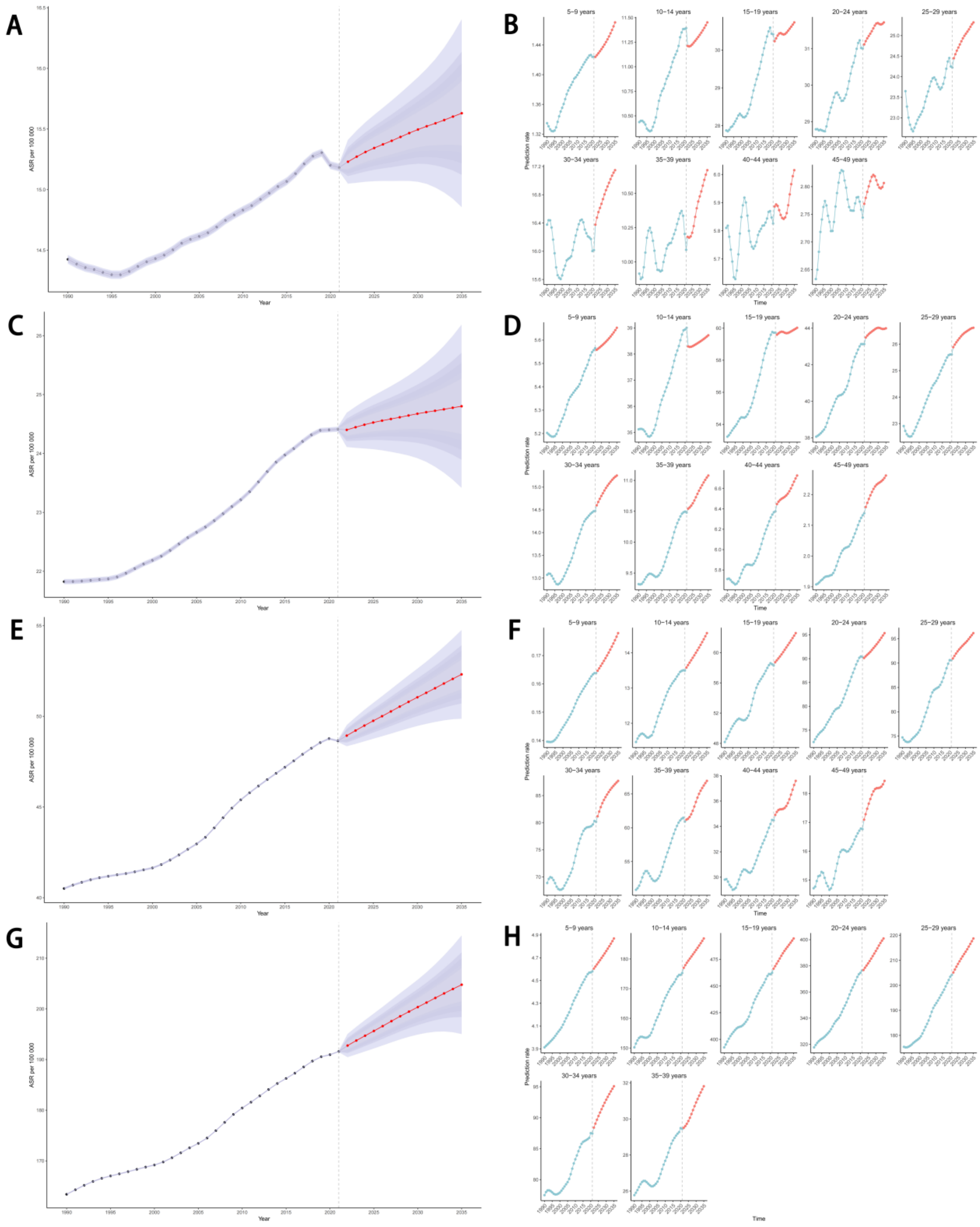

Fig. S8 :Projections of anorexia nervosa age-standardized DALYs rate (A-B)and ASIR (C-D) by 2035 based on the BAPC model. Projections of bulimia nervosa age-standardized DALYs rate (E-F)and ASIR (G-H) by 2035 based on the BAPC model. DALYs, disability-adjusted life years; ASIR, age-standardized incidence rate; BAPC, Bayesian Age-Period-Cohort.

Location	ASDR (95%UI) in 2021(per 100,000 population)	Location	ASDR (95%UI) in 2021(per 100,000 population)	Location	ASDR (95%UI) in 2021(per 100,000 population)	Location	ASDR (95%UI) in 2021(per 100,000 population)
Mongolia	9.16e-04(2.37e-04,2.74e-03)	Slovakia	4.21e-04(1.08e-04,8.63e-04)	Switzerland	4.34e-06(4.02e-07,1.27e-05)	Luxembourg	1.58e-02(1.18e-02,2.13e-02)
Australia	1.10e-03(2.23e-04,5.08e-03)	Russian Federation	5.54e-02(4.30e-02,6.91e-02)	Guatemala	6.84e-04(5.00e-04,9.35e-04)	Paraguay	9.58e-04(3.23e-04,2.00e-03)
Tajikistan	9.58e-03(4.15e-03,1.85e-02)	Hungary	1.90e-02(1.49e-02,2.41e-02)	Greece	1.90e-02(1.49e-02,2.41e-02)	tedIvoire	1.70e-05(3.34e-06,3.62e-05)
Togo	2.10e-03(4.95e-04,6.35e-03)	Seychelles	2.96e-05(2.78e-06,9.28e-05)	Andorra	4.03e-03(1.64e-03,8.01e-03)	Qatar	2.10e-05(3.93e-06,5.92e-05)
Turkmenistan	4.71e-03(2.11e-03,6.16e-03)	Niger	4.84e-05(2.59e-05,7.11e-05)	Lebanon	2.96e-05(2.78e-06,9.28e-05)	Suriname	2.83e-05(8.23e-06,5.24e-05)
Belarus	7.54e-04(1.42e-04,1.69e-03)	Kenya	5.72e-05(1.13e-05,9.88e-05)	Zambia	3.71e-05(1.44e-05,6.56e-05)	Sri Lanka	4.47e-04(1.20e-04,8.97e-04)
Malta	7.01e-05(1.56e-05,1.21e-04)	Netherlands	3.03e-02(2.34e-02,3.88e-02)	South Africa	4.84e-05(2.59e-05,7.11e-05)	Saint Vincent and the Grenadines	1.83e-05(1.18e-05,2.25e-05)
Norway	2.03e-03(1.23e-03,2.83e-03)	Barbados	4.14e-05(1.82e-05,7.54e-05)	Jordan	2.16e-05(2.68e-06,6.08e-05)	Cameroon	3.01e-05(1.09e-05,5.89e-05)
Democratic People's Republic of Korea	2.57e-04(8.45e-05,5.55e-04)	El Salvador	7.50e-06(1.16e-06,1.78e-05)	Samoa	5.72e-05(1.13e-05,9.88e-05)	Guam	2.23e-05(4.86e-06,2.36e-05)
Chad	8.84e-06(1.60e-06,2.41e-05)	Ghana	5.71e-06(1.31e-06,1.16e-05)	Costa Rica	4.97e-03(3.75e-03,6.51e-03)	Mali	9.96e-06(1.65e-06,1.72e-05)
Kiribati	7.53e-03(5.81e-03,9.52e-03)	Armenia	5.27e-05(2.20e-05,1.05e-04)	Austria	3.03e-02(2.34e-02,3.88e-02)	Bermuda	4.10e-05(5.22e-06,1.23e-04)
Marshall Islands	2.56e-04(9.46e-05,4.74e-04	Dominica	1.56e-05(1.64e-06,3.68e-0	Montenegro	7.62e-04(1.80e-04,2.14e-03	Saint Kitts and	4.06e-05(1.15e-05,1.13

	)		5)		)	Nevis	e-04)
China	3.44e-05(9.28e-06,8.47e-05)	Myanmar	3.10e-05(1.23e-05,6.17e-05)	Eswatini	4.14e-05(1.82e-05,7.54e-05)	Republic of Moldova	4.17e-03(2.54e-03,6.63e-03)
Bosnia and Herzegovina	7.99e-05(3.20e-05,1.33e-04)	Latvia	1.05e-03(7.74e-04,1.40e-03)	Japan	3.91e-02(3.34e-02,4.58e-02)	Guinea-Bissau	1.70e-05(4.99e-06,2.86e-05)
Congo	2.27e-03(9.46e-04,4.33e-03)	Uzbekistan	2.18e-05(7.79e-06,5.39e-05)	Yemen	7.50e-06(1.16e-06,1.78e-05)	Liberia	1.53e-05(3.90e-06,2.82e-05)
Albania	1.44e-05(1.76e-06,2.77e-05)	United States of America	5.97e-05(1.92e-05,1.65e-04)	Brazil	2.04e-03(1.66e-03,2.36e-03)	San Marino	1.29e-05(1.28e-06,1.06e-05)
United Republic of Tanzania	1.52e-04(2.33e-05,4.55e-04)	Mexico	1.19e-05(2.99e-06,2.18e-05)	Ethiopia	5.71e-06(1.31e-06,1.16e-05)	Sao Tome and Principe	3.09e-05(7.54e-06,8.33e-05)
Bolivia (Plurinational State of)	4.57e-03(2.70e-03,7.30e-03)	Somalia	5.20e-06(5.81e-07,1.25e-05)	Singapore	2.95e-03(2.16e-03,4.03e-03)	Fiji	5.95e-05(1.92e-05,8.40e-05)
Monaco	1.16e-05(7.22e-06,1.23e-05)	Micronesia (Federated States of)	5.51e-04(1.36e-04,1.08e-03)	Gabon	5.27e-05(2.20e-05,1.05e-04)	Ukraine	1.53e-03(6.54e-04,3.04e-03)
Spain	4.71e-05(9.24e-06,7.82e-05)	Lithuania	1.33e-05(7.72e-06,1.62e-05)	Taiwan (Province of China)	2.47e-04(1.23e-04,3.08e-04)	Kazakhstan	1.67e-03(4.22e-04,3.36e-03)
Belgium	1.96e-05(1.29e-06,6.70e-05)	Lesotho	1.02e-02(8.42e-03,1.22e-02)	Senegal	1.56e-05(1.64e-06,3.68e-05)	Czechia	1.98e-02(1.10e-02,3.24e-02)
Peru	4.05e-05(1.07e-05,1.06e-04)	Cyprus	3.39e-05(1.10e-05,8.84e-05)	Botswana	3.16e-05(1.46e-05,5.50e-05)	Nigeria	2.83e-05(8.39e-06,5.75e-05)
Democratic Republic of the Congo	1.17e-02(1.03e-02,1.30e-02)	Pakistan	2.17e-05(5.82e-06,4.07e-05)	Chile	2.33e-03(1.84e-03,2.92e-03)	Republic of Korea	4.83e-04(2.83e-04,7.76e-04)
United Arab Emirates	1.44e-03(1.02e-03,2.04e-03)	Portugal	2.70e-03(8.76e-04,4.82e-03)	Panama	1.05e-03(7.74e-04,1.40e-03)	Tokelau	9.74e-05(2.89e-05,1.51e-04)

Tunisia	5.32e-05(3.66e-05,6.74e-05)	Burundi	3.39e-05(1.14e-05,6.21e-05)	Haiti	1.28e-05(2.72e-06,3.03e-05)	Palau	9.21e-05(2.72e-05,1.39e-04)
Nauru	3.67e-04(2.32e-04,4.82e-04)	Papua New Guinea	8.59e-03(6.57e-03,1.12e-02)	Belize	2.18e-05(7.79e-06,5.39e-05)	Iraq	2.67e-05(9.35e-06,5.72e-05)
Indonesia	9.36e-06(3.17e-06,1.21e-05)	United States Virgin Islands	1.58e-02(1.18e-02,2.13e-02)	Trinidad and Tobago	5.97e-05(1.92e-05,1.65e-04)	Honduras	7.71e-04(2.96e-04,1.66e-03)
Turkey	1.15e-02(6.44e-03,2.01e-02)	Uruguay	1.70e-05(3.34e-06,3.62e-05)	Angola	3.10e-05(1.23e-05,6.17e-05)	Namibia	3.35e-05(1.54e-05,6.44e-05)
Guinea	2.73e-06(1.73e-07,8.50e-06)	Greenland	2.83e-05(8.23e-06,5.24e-05)	Djibouti	1.83e-05(3.35e-06,3.34e-05)	Egypt	2.39e-05(4.17e-06,7.17e-05)
Solomon Islands	3.80e-02(3.01e-02,4.73e-02)	Brunei Darussalam	1.83e-05(1.18e-05,2.25e-05)	Sierra Leone	1.19e-05(2.99e-06,2.18e-05)	Sudan	9.97e-06(8.64e-07,3.42e-05)
Cambodia	8.22e-04(3.21e-04,1.57e-03)	Romania	3.01e-05(1.09e-05,5.89e-05)	Northern Mariana Islands	3.27e-05(6.85e-06,3.81e-05)	Bangladesh	1.54e-04(1.83e-05,7.82e-04)
Estonia	3.91e-05(1.74e-05,7.27e-05)	Algeria	9.96e-06(1.65e-06,1.72e-05)	Burkina Faso	5.20e-06(5.81e-07,1.25e-05)	Canada	1.41e-02(1.09e-02,1.82e-02)
North Macedonia	3.70e-05(9.15e-06,7.64e-05)	Kyrgyzstan	4.17e-03(2.54e-03,6.63e-03)	Denmark	2.79e-02(2.09e-02,3.60e-02)	Finland	2.82e-02(2.18e-02,3.68e-02)
Cuba	1.05e-03(8.18e-04,1.33e-03)	Israel	1.29e-05(1.28e-06,1.06e-05)	Central African Republic	1.05e-05(3.39e-06,1.79e-05)	Bhutan	7.55e-05(3.57e-06,4.19e-04)
Uganda	1.50e-03(5.19e-04,3.71e-03)	Azerbaijan	5.95e-05(1.92e-05,8.40e-05)	Nicaragua	5.51e-04(1.36e-04,1.08e-03)	Kuwait	1.50e-04(1.04e-04,2.12e-04)
Cabo Verde	3.45e-06(1.90e-07,7.28e-06)	Rwanda	1.67e-03(4.22e-04,3.36e-03)	Palestine	1.59e-05(1.11e-06,4.39e-05)	Eritrea	1.61e-05(5.27e-06,2.72e-05)
Iran (Islamic Republic of)	9.81e-03(6.23e-03,1.47e-02)	India	1.98e-02(1.10e-02,3.24e-02)	Saint Lucia	1.33e-05(7.72e-06,1.62e-05)	Thailand	5.89e-04(1.93e-04,1.23e-03)
Tonga	1.63e-02(8.37e-03,2.62e-02)	Bulgaria	4.83e-04(2.83e-04,7.76e-04)	Mozambique	1.56e-05(3.21e-06,3.17e-05)	Oman	2.17e-05(7.06e-06,4.48e-05)

Colombia	4.57e-03(3.46e-03,5.72e-03)	Ecuador	9.21e-05(2.72e-05,1.39e-04)	Italy	1.02e-02(8.42e-03,1.22e-02)	Cook Islands	6.21e-05(1.54e-05,1.15e-04)
Syrian Arab Republic	4.50e-06(1.44e-07,1.57e-05)	Nepal	7.71e-04(2.96e-04,1.66e-03)	Lao People's Democratic Republic	1.52e-04(2.33e-05,4.55e-04)	Mauritius	1.16e-05(8.04e-06,1.42e-05)
Comoros	1.42e-03(5.91e-04,2.55e-03)	Serbia	2.39e-05(4.17e-06,7.17e-05)	Madagascar	5.23e-06(1.90e-07,1.58e-05)	Venezuela (Bolivarian Republic of)	9.79e-04(5.95e-04,1.60e-03)
Antigua and Barbuda	9.77e-03(5.05e-03,1.69e-02)	Jamaica	1.54e-04(1.83e-05,7.82e-04)	Grenada	3.39e-05(1.10e-05,8.84e-05)	Libya	6.03e-05(9.98e-06,1.61e-04)
Puerto Rico	8.46e-04(2.83e-04,1.79e-03)	Guyana	2.82e-02(2.18e-02,3.68e-02)	Gambia	2.05e-05(4.60e-06,5.05e-05)	Benin	1.07e-05(1.95e-06,1.96e-05)
United Kingdom	8.01e-03(6.15e-03,1.02e-02)	Bahrain	1.50e-04(1.04e-04,2.12e-04)	Malawi	2.17e-05(5.82e-06,4.07e-05)	Morocco	2.04e-05(4.43e-06,4.90e-05)
Slovenia	2.27e-05(4.83e-06,5.51e-05)	Croatia	5.89e-04(1.93e-04,1.23e-03)	American Samoa	4.26e-05(1.06e-05,4.92e-05)	France	2.22e-02(1.71e-02,2.86e-02)
Argentina	4.27e-03(2.67e-03,6.58e-03)	Equatorial Guinea	6.21e-05(1.54e-05,1.15e-04)	Georgia	2.70e-03(8.76e-04,4.82e-03)	Saudi Arabia	3.31e-04(1.05e-04,6.49e-04)
Afghanistan	1.00e-04(9.15e-06,5.24e-04)	Sweden	9.79e-04(5.95e-04,1.60e-03)	Malaysia	4.66e-04(9.20e-05,1.04e-03)	Ireland	1.97e-02(1.44e-02,2.59e-02)
New Zealand	2.97e-05(8.38e-06,8.36e-05)	Mauritania	1.07e-05(1.95e-06,1.96e-05)	Zimbabwe	3.39e-05(1.14e-05,6.21e-05)	Timor-Leste	1.26e-04(2.75e-05,2.79e-04)
Germany	2.40e-05(6.93e-06,5.20e-05)	Vanuatu	5.53e-05(1.17e-05,8.72e-05)	Niue	1.12e-04(3.25e-05,2.12e-04)	Tuvalu	5.53e-05(1.17e-05,8.72e-05)
Philippines	6.49e-05(2.73e-05,1.32e-04)	Viet Nam	3.31e-04(1.05e-04,6.49e-04)	Iceland	8.59e-03(6.57e-03,1.12e-02)	Poland	1.34e-02(1.00e-02,1.75e-02)
Bahamas	2.09e-05(8.22e-06,3.65e-05)	Dominican Republic	1.26e-04(2.75e-05,2.79e-04)	Maldives	1.79e-04(1.46e-05,4.99e-04)	South Sudan	4.34e-06(4.02e-07,1.27e-05)

Table S1:The ASDR of eating disorders across 204 countries and territories in 2021. UI, uncertainty intervals; ASDR, age-standardized death rate

Location	age-standardized DALYs rate (95%UI) in 2021(per 100,000 population)	Location	age-standardized DALYs rate (95%UI) in 2021(per 100,000 population)	Location	age-standardized DALYs rate (95%UI) in 2021(per 100,000 population)	Location	age-standardized DALYs rate (95%UI) in 2021(per 100,000 population)
Thailand	33.68(19.53,54.21)	Uruguay	81.37(48.97,127.81)	Taiwan (Province of China)	42.24(25.05,66.38)	Turkmenistan	40.88(24.47,64.39)
Guinea-Bissau	24.22(14.41,38.45)	Singapore	109.19(67.88,167.49)	Burkina Faso	24.88(14.99,39.44)	Madagascar	23.88(14.38,37.59)
Vanuatu	19.9(12.04,31.51)	Germany	97.77(62.56,147.25)	Marshall Islands	21.01(12.96,33.36)	South Africa	42.89(26.29,68.54)
Northern Mariana Islands	38.29(23.55,60.58)	Saint Vincent and the Grenadines	51.95(31.44,80.78)	South Sudan	25.07(15.1,39.4)	United States Virgin Islands	82.88(49.02,131.57)
Slovakia	45.15(26.82,70.21)	Sweden	115.12(70.8,180.47)	Nicaragua	40.1(23.99,63.52)	Solomon Islands	18.4(11.27,28.75)
Sudan	34.79(20.54,55.64)	Bulgaria	40.37(24.2,63.61)	Kyrgyzstan	26.64(16.07,41.72)	Spain	127.59(77.67,195.83)
Malaysia	38.27(22.78,61.2)	Syrian Arab Republic	37.18(22.32,57.78)	Bangladesh	31.1(18.64,48.79)	Togo	24.13(14.3,38.24)
Micronesia (Federated States	20.63(12.37,32.47)	Saint Lucia	53.44(31.18,84.81)	Iceland	103.75(64.73,156.68)	Mozambique	22.31(14.11,34.86)

of)							
Guam	45.59(27.02,73.2)	Bahrain	64.19(38.57,102.83)	Chad	25.39(15.54,40.26)	Democratic Republic of the Congo	19.63(11.83,30.48)
Albania	34.55(21.12,54.46)	American Samoa	31.34(18.85,50.1)	Viet Nam	26.05(15.61,42)	Suriname	56.39(34.61,88.46)
Slovenia	46.1(27.43,72.89)	Pakistan	33.54(19.9,53.13)	Palau	30.92(18.53,48.12)	Venezuela (Bolivarian Republic of)	52.32(30.46,83.43)
Democratic People's Republic of Korea	16.73(10.35,26.12)	Comoros	27.26(16.34,43.68)	Latvia	45.35(27.4,70.4)	Serbia	37.9(23.33,59.64)
Timor-Leste	25.32(15.31,40.58)	United Republic of Tanzania	28.43(17.19,45.62)	Maldives	31.75(18.97,51.16)	Mexico	58.26(34.65,93.59)
Japan	101.69(62.97,155.36)	Azerbaijan	38.62(23.49,59.83)	Djibouti	30.53(18.56,48.92)	Paraguay	52.72(31.68,80.81)
Seychelles	37.93(22.72,59.41)	Cameroon	29.28(17.83,46.61)	Papua New Guinea	21.43(12.76,34.44)	Bahamas	71.13(41.71,112.05)
Norway	109.85(67.22,170.08)	Uganda	25.73(15.17,41.04)	Liberia	20.88(12.63,33.34)	Iran (Islamic Republic of)	61.25(37.23,96.62)
Brunei Darussalam	107.02(65.33,163.71)	Nepal	28.46(17.81,45.75)	Bosnia and Herzegovina	35.2(21.21,55.79)	Yemen	29.59(17.5,46.99)
Denmar	103.58(63.45,1	Libya	43.73(26.25,67.	Jordan	44.04(25.73,70.	Belgiu	101.28(61.94,1

k	58.26)		78)		4)	m	50.94)
United States of America	98.9(60.08,152.96)	Guatemala	45.76(27.23,72.99)	Palestine	35.9(21.49,56.86)	Afghanistan	27.81(17,42.96)
Finland	111.74(69.19,167.38)	Mauritania	30.74(18.41,48.68)	Rwanda	25.78(15.46,40.89)	Congo	33.72(20.33,53.48)
Botswana	46.54(28.12,74.26)	Niger	20.83(12.24,33.36)	Dominica	50.84(30.15,79.48)	Republic of Moldova	32.33(19.97,49.34)
Kenya	29.73(17.89,47.23)	Brazil	54.59(33.25,86.02)	Iraq	51.04(29.72,80.48)	Greenland	114.95(70.05,177.05)
Mali	26.01(15.82,42.08)	Barbados	57.31(33.88,90.96)	Mongolia	35.53(21.68,55.87)	Malta	98.08(59.97,148.91)
Myanmar	25.47(15.61,39.8)	Fiji	27.9(16.97,44.64)	Niue	30.06(18.18,47.45)	Cyprus	92.56(56.59,139.3)
Qatar	84.19(50.87,131.22)	Luxembourg	132.63(79.76,201.18)	Republic of Korea	85.55(51.19,132.01)	Cuba	47.86(28.81,76.54)
Haiti	30.3(17.89,47.55)	Trinidad and Tobago	72.77(43.01,116.84)	Chile	82.53(49.54,129.95)	Cook Islands	40.2(24.32,62.55)
Bhutan	38.8(22.94,61.64)	Georgia	34.3(20.78,53.93)	Montenegro	39.04(23.51,59.81)	Sri Lanka	30.75(19.07,48.21)
Samoa	24.13(14.72,38.15)	Kiribati	18.2(11.17,28.69)	Argentina	77.53(46.59,122.95)	Eritrea	24.37(14.67,38.28)
Portugal	90.07(55.69,138.7)	Saudi Arabia	62.77(37.81,97.61)	North Macedonia	36.22(22.06,56.01)	Greece	99.89(61.54,152.33)
Jamaica	47.99(28.84,77.45)	Honduras	40.08(23.45,63.81)	Senegal	29.08(17.14,45.8)	Estonia	46.97(28.74,73.65)
Algeria	49.66(30.32,78.11)	Nauru	29.89(18.01,47.64)	Lesotho	28.69(17.89,45.71)	Philippines	27.07(16.37,42.86)
Cotedivoire	30.62(18.33,48.95)	Colombia	54.11(32.37,84.37)	Russian Federation	45.66(27.7,71.02)	Grenada	54.94(32.48,87.15)
Puerto Rico	75.5(44.56,118.63)	Lao People's Democratic	25.96(15.5,40.87)	Angola	35.84(21.59,57.34)	Gambia	26.09(15.93,41.78)

		Republic					
Poland	49.06(30.43,75.58)	Burundi	19.03(11.33,30.48)	Central African Republic	19.27(11.6,30.7)	Netherlands	87.41(54.91,129.82)
El Salvador	46.3(27.7,73.11)	Guyana	49.11(29.26,77.7)	Namibia	41.73(25.53,65.27)	Malawi	21.84(13.4,34.35)
Somalia	12.35(7.3,19.59)	Hungary	47.62(28.59,74.71)	Croatia	44.29(27.49,68.84)	Uzbekistan	32.42(19.92,51.58)
Tajikistan	24.47(14.68,38.41)	Kazakhstan	43.93(26.39,69.11)	Bermuda	88.69(52.08,138.32)	Morocco	42.57(25.44,67.77)
Benin	27.07(16.57,42.29)	Ethiopia	24.8(14.97,39.69)	France	108.5(67.22,164.86)	Canada	95.97(59.03,145.78)
Costa Rica	56.83(33.66,91.23)	Australia	225.9(145.77,330.9)	Kuwait	78.9(47.5,123.72)	Tonga	24.46(14.3,38.49)
Armenia	33.45(20.68,51.85)	Turkey	62.41(37.08,99.76)	Israel	82.47(49.89,125.18)	Eswatini	39.1(23.66,61.24)
Tuvalu	21.28(12.6,33.58)	New Zealand	132.69(80.91,207.49)	Zimbabwe	26.57(15.55,41.71)	Mauritius	35.48(21.08,57.78)
Belarus	40.88(25.11,63.76)	Lithuania	46.78(28.19,73.04)	Bolivia (Plurinational State of)	57.28(33.62,91.99)	Switzerland	103.5(65.35,152.66)
Antigua and Barbuda	65.39(39.02,105.01)	Gabon	46.38(27.65,72.75)	Saint Kitts and Nevis	68.08(39.64,107.56)	China	29.6(17.94,46.99)
Oman	62.86(37.04,96.61)	Nigeria	34.1(20.35,54.65)	Lebanon	48.82(28.76,76.56)	Equatorial Guinea	50.37(30.15,77.94)
Peru	69.26(39.26,110.5)	Belize	47.68(28.16,76.28)	Italy	124.35(76.45,191.84)	Sao Tome and Principe	29.49(17.71,46.33)
Ukraine	33.87(20.56,52.	Egypt	47.56(27.62,75.	Andor	116.94(71.35,1	Zambia	30.44(18.67,48.

	15)		57)	ra	75.1)		23)
India	34.94(21.19,55.75)	Ireland	108.43(67.33,167.13)	Panama	64(37.39,100.75)	Tunisia	47.09(28.69,74.04)
Sierra Leone	23.12(14.32,36.3)	Monaco	193.35(116.62,295.57)	United Arab Emirates	70.21(42.94,109.89)	San Marino	107.55(65.61,163.89)
Dominican Republic	58.4(34.8,93.16)	United Kingdom	102.38(62.04,158.35)	Ecuador	65.67(38.19,105.36)	Cabo Verde	35.9(21.87,57.63)
Cambodia	22.05(12.83,34.99)	Indonesia	29.25(17.78,46.3)	Ghana	33.4(20.72,52.3)	Guinea	26.26(15.38,41.73)
Austria	115.6(70.89,177.17)	Czechia	48.89(30.43,74.56)	Romania	41.38(25.17,64.36)	Tokelau	25.93(15.36,41.08)

Table S2:The age-standardized DALYs rate of eating disorders across 204 countries and territories in 2021. UI, uncertainty intervals; DALYs, disability-adjusted life years.

**Location**	**ASIR (95%UI) in 2021(per 100,000 population)**	**Location**	**ASIR (95%UI) in 2021(per 100,000 population)**	**Location**	**ASIR (95%UI) in 2021(per 100,000 population)**	**Location**	**ASIR (95%UI) in 2021(per 100,000 population)**
China	115.98(78.58,168.45)	Republic of Moldova	99.91(69.98,140.08)	Bolivia (Plurinational State of)	129.21(87.21,182.39)	Somalia	59.8(40.79,87.72)
Indonesia	110.01(75.58,159.65)	Andorra	172.87(123.32,233.77)	Peru	142.25(97.31,200.58)	Mauritius	105.59(72.72,149.23)
Malaysia	110.72(76.89,157.27)	Austria	165.29(117.51,224.77)	Colombia	136.56(94.25,192.74)	Uganda	87.16(59.66,124.29)
Myanmar	86.85(59.59,122.75)	Denmark	169.76(120.26,229.11)	El Salvador	122.71(83.78,175.79)	Gabon	118.53(81.52,167.97)
Thailand	101.55(70.26,143.79)	Greece	155.54(111.41,206.96)	Costa Rica	138.42(93.85,194.81)	Eritrea	85.83(58.26,122.89)
Viet Nam	89.6(61.66,127.49)	Japan	206.89(147.04,283.96)	Guatemala	122.55(83.37,173.91)	Equatorial Guinea	132.21(89.3,188.45)
Fiji	93.44(64.25,132.65)	Republic of Korea	169.54(117.67,233.69)	Nicaragua	115.21(78.33,163.63)	Burkina Faso	84.51(57.85,121.73)
Tonga	85.87(59.12,121.25)	Belgium	158(111.94,211.82)	Mexico	162.18(108.38,229.98)	Benin	89.41(62.29,126.25)
Turkmenistan	117.89(81.41,167.35)	Singapore	191.3(134.55,260.49)	Algeria	136.17(94.4,187.73)	Cabo Verde	106.37(71.88,150.95)

Kazakhstan	120.56(83.93,171.44)	Cyprus	151.3(107.85,201.74)	Venezuela (Bolivarian Republic of)	130.17(90.26,179.31)	Zambia	95.65(64.96,136.75)
Mongolia	107.11(73.97,149.09)	Finland	170.7(121.92,223.52)	Egypt	133.78(92.52,188.47)	United Republic of Tanzania	91.28(62.19,127.94)
Kyrgyzstan	91.55(64.49,129.42)	France	160.75(112.13,213.66)	Iran (Islamic Republic of)	181.52(125.76,251.51)	Namibia	113.55(78.14,159.89)
Cambodia	81.58(55.89,117.48)	Germany	150.88(107.54,200.74)	Panama	148.26(99.71,207.05)	Lesotho	93.9(63.88,133.53)
Lao People's Democratic Republic	89.29(60.76,126.19)	Russian Federation	142.14(99.25,201.93)	Bahrain	169.99(115.99,233.88)	South Africa	136.57(92.92,197.83)
Democratic People's Republic of Korea	71.05(48.36,101.64)	Ukraine	121.91(83.97,172.44)	Paraguay	130.56(91.12,183.31)	Zimbabwe	88.43(60.89,126.59)
Taiwan (Province of China)	117.69(80.18,164.36)	Australia	338.06(242.51,458.81)	Brazil	154.81(105.19,218.28)	Cameroon	94.11(65.73,133.5)
Maldives	105.41(73.08,150.7)	New Zealand	286.76(194.61,402.37)	Lebanon	136.88(94.63,189.93)	Chad	86.21(58.97,123.1)
Micronesia (Federated States of)	79.34(53.13,113.14)	Iceland	161.23(114.33,220.64)	Palestine	114.7(79.14,159.68)	Gambia	87.86(59.95,125.63)
Brunei Darussalam	188.76(134.43,258.7)	Ireland	166.8(118.48,225.27)	Qatar	203.91(140.48,281.95)	Guinea	87.41(59.87,124.87)
Philippines	104.92(72.19,152.5)	Sweden	380.71(246.62,559.27)	Morocco	125.92(87.18,175.48)	Ghana	100.48(68.48,140.34)
Timor-Leste	87.57(59.52,125.9)	Netherlands	114.88(82.81,154.21)	Libya	127.51(87.89,176.38)	Cotedivoire	97.32(66.57,141.01)
Marshall Islands	79.98(54.42,115.25)	Italy	155.92(110.78,210.39)	Syrian Arab Republic	111.87(78.89,155.95)	Botswana	121.04(83.31,169.56)
Papua New Guinea	81.46(55.38,117.39)	Norway	204.38(144.09,278.22)	Oman	165.08(115.66,229.63)	Mali	87.93(60.6,126.65)
Samoa	86.23(58.89,1	Portugal	149.82(106.6	Kuwait	175.51(124.2	Guinea-	84.01(57.3,11

	21.03)		2,199.7)		9,239.49)	Bissau	8.22)
Armenia	104.68(72.59, 148.49)	Israel	123.51(87.84, 166.31)	Tunisia	132.56(91.35, 186.04)	Eswatini	110.16(76.14, 157.11)
Solomon Islands	74.12(50.35,105.47)	Switzerland	156.8(111.59, 211.8)	Turkey	148.09(103.19,207.55)	Mauritania	95.61(65.16,135.85)
Kiribati	72.95(50.35,105.45)	Luxemburg	182.42(131.36,245)	Saudi Arabia	164.13(113.81,228.8)	Liberia	79.18(53.64,112.94)
Sri Lanka	97.2(66.68,136.37)	Saint Vincent and the Grenadines	128.73(86.43, 183.23)	United Arab Emirates	175.89(123.44,241.05)	Senegal	94.62(64.86,135.79)
Azerbaijan	112.5(77.64,155.06)	Malta	155.89(109.55,210.44)	Bangladesh	96.29(66.87,137.17)	American Samoa	99.34(68.07,139.38)
Georgia	106.04(73.66, 151.62)	Bahamas	151.05(102.85,212.42)	Afghanistan	100.97(69.19, 140.59)	Sierra Leone	82.29(56.02,117.07)
Tajikistan	87.93(61.62,125.27)	Barbados	134.14(92.7,186.98)	Bhutan	111.2(76.02,156.38)	Togo	84.6(57.35,121.68)
Croatia	123.24(85.98, 172.78)	United States of America	211.13(142.94,298.87)	Djibouti	98.73(66.81,141.74)	Guam	122.74(83.99, 172.92)
Vanuatu	77.37(51.71,112.27)	United Kingdom	175.44(123.42,239.29)	Jordan	130.47(89.63, 182.93)	Bermuda	169.48(117.36,232.94)
Bosnia and Herzegovina	110.45(78.01, 154.24)	Belize	122.31(82.45, 170.08)	Nepal	91.89(62.88,131.16)	Saint Kitts and Nevis	148.19(100.79,212.58)
Albania	111.28(77.49, 156.38)	Cuba	124.11(84.99, 175.96)	India	122.13(82.47, 176.56)	Monaco	230.49(166.08,309.21)
Uzbekistan	102.92(70.86, 145.87)	Dominica	128.12(87.64, 182.26)	Iraq	139.92(97.33, 192.48)	Palau	102.88(68.22, 147.7)
Bulgaria	118.87(83.3,165.29)	Canada	181.51(127.66,247.05)	Comoros	90.55(61.88,127.95)	Cook Islands	110.67(75.07, 155.4)
Czechia	130.15(89.3,183.15)	Grenada	133.45(90.95, 188.57)	Ethiopia	99.2(66.94,145.44)	Sao Tome and Principe	94.4(63.96,134.88)
Hungary	127.61(88.5,178.76)	Guyana	124.91(84.63, 177.49)	Burundi	73.73(50.6,104.07)	Nigeria	116.68(79.44, 169.14)
Poland	149.67(104.83,212.83)	Haiti	96.5(65.48,138.21)	Congo	100.86(68.05, 144.32)	Niger	77.91(53.42,110.09)
Serbia	116.07(79.78, 164.48)	Dominican Republic	137.14(93.09, 195.24)	Democratic Republic of the	76.61(52.52,109.58)	South Sudan	85.99(58.18,122.6)

				Congo			
Estonia	125.46(87.55,177.59)	Antigua and Barbuda	144.46(99.27,201.65)	Pakistan	119.54(81.2,172.22)	Niue	97.78(66.73,139.17)
Spain	228.78(161.36,311.4)	Chile	166.4(115.76,226.07)	Yemen	103.19(70.62,144.54)	Nauru	97(66.27,137.13)
North Macedonia	112.72(78.53,159.26)	Uruguay	163.87(112.27,221.31)	Kenya	109.34(74.53,158.61)	Tuvalu	81.75(56.03,116.49)
Montenegro	117.2(80.69,166.61)	Argentina	158.94(109.51,218.28)	Central African Republic	74.44(51.25,106.4)	Greenland	195.63(139.31,264.85)
Romania	122.37(84.55,169.22)	Suriname	134.32(92.06,190.32)	Angola	104.31(71.11,146.9)	Tokelau	90.52(62.3,129.31)
Slovakia	126.87(87.72,175.08)	Jamaica	122.97(85.36,173.49)	Madagascar	83.78(58.04,118.73)	San Marino	164.77(119.11,221.47)
Slovenia	128.93(90.44,180.6)	Saint Lucia	131.08(88.84,181.96)	Malawi	78.9(55,111.98)	Puerto Rico	155.74(105.56,219.82)
Lithuania	124.11(86.6,172.48)	Trinidad and Tobago	154.34(105.22,217.33)	Rwanda	87.09(59.16,124.21)	Northern Mariana Islands	111.4(76.86,157.81)
Latvia	122.98(85.23,171.12)	Ecuador	137.82(93.38,191.73)	Mozambique	80.14(55.22,113.34)	United States Virgin Islands	163.65(109.75,230.96)
Belarus	116.12(80.77,161.86)	Honduras	113.71(78.43,160.76)	Seychelles	111.19(77.52,158.09)	Sudan	112.18(78.09,155.48)

Table S3:The ASIR of eating disorders across 204 countries and territories in 2021. UI, uncertainty intervals; ASIR, age-standardized incidence rate.

**Location**	**age-standardized DALYs rate (95%UI) in 2021(per 100,000 population)**	**Location**	**age-standardized DALYs rate (95%UI) in 2021(per 100,000 population)**	**Location**	**age-standardized DALYs rate (95%UI) in 2021(per 100,000 population)**	**Location**	**age-standardized DALYs rate (95%UI) in 2021(per 100,000 population)**
Thailand	8.37(5.18,13.9)	Uruguay	18.19(10.54,29.76)	Marshall Islands	5.28(3.1,8.37)	Turkmenistan	10.92(6.39,17.19)
Guinea-Bissau	5.66(3.16,9.36)	Singapore	31.1(18.16,49.72)	South Sudan	5.79(3.25,9.37)	Madagascar	5.6(3.28,8.98)

Vanuatu	5.07(2.99,8.19)	Germany	37.21(22.61,57.78)	Nicaragua	8(4.76,12.89)	South Africa	8.87(5.34,14.11)
Slovakia	11.43(6.84,18.17)	Saint Vincent and the Grenadines	9.5(5.85,15.52)	Kyrgyzstan	7.58(4.49,12.29)	United States Virgin Islands	13.97(8.22,22.47)
Sudan	6.68(3.9,10.89)	Sweden	30(18.86,47.23)	Bangladesh	7.03(4.04,11.19)	Spain	36.89(21.36,58.31)
Malaysia	9.28(5.6,15.03)	Bulgaria	10.48(6.22,16.93)	Iceland	28.63(16.65,45.39)	Togo	5.63(3.17,8.99)
Micronesia (Federated States of)	5.19(2.98,8.55)	Syrian Arab Republic	7.1(4.12,11.23)	Chad	5.88(3.57,9.68)	Mozambique	5.33(3.23,8.54)
Albania	8.91(5.24,14.54)	Saint Lucia	9.62(5.71,15.47)	Viet Nam	6.68(3.86,10.74)	Democratic Republic of the Congo	4.73(2.8,8.02)
Slovenia	11.59(6.85,18.68)	Bahrain	11.03(6.65,18.16)	Palau	7.12(4.23,11.43)	Suriname	10.09(5.88,16.8)
Democratic People's Republic of Korea	4.88(2.87,7.81)	American Samoa	7.36(4.2,12.21)	Latvia	13.04(7.84,21.21)	Venezuela (Bolivarian Republic of)	9.9(5.75,16.08)
Timor-Leste	6.45(3.86,10.24)	Comoros	6.26(3.74,10.06)	Maldives	7.55(4.35,12.22)	Serbia	10.05(5.85,16.02)
Japan	38.24(24.16,58.87)	United Republic of Tanzania	6.5(3.76,10.73)	Papua New Guinea	5.34(3.13,8.62)	Mexico	10.3(6.31,16.68)
Angola	7.86(4.69,12.65)	Cameroon	6.65(3.89,10.78)	Liberia	5(2.92,8.38)	Paraguay	15.02(8.79,23.91)
Seychelles	9.24(5.49,15.72)	Uganda	5.99(3.45,9.87)	Djibouti	6.72(4.04,10.84)	Bahamas	12.34(7.29,19.77)
Bhutan	8.35(4.85,13.59	Nepal	6.51(3.75,10.49	Bosni	9.48(5.55,14.93	Iran	12.07(7.16,19.4

	)		)	a and Herzegovina	)	(Islamic Republic of)	8)
Norway	24.56(15.27,38.65)	Libya	8.1(4.69,13.59)	Jordan	8.09(4.8,12.99)	Yemen	5.85(3.38,9.75)
Brunei Darussalam	34.16(20.14,53.82)	Guatemala	8.92(5.17,14.37)	Palestine	6.85(3.89,10.78)	Belgium	32.73(20.36,50.96)
Denmark	29.81(18.23,46.73)	Mauritania	6.88(4.1,11.24)	Rwanda	6(3.46,10)	Afghanistan	5.58(3.24,8.92)
United States of America	30.04(18.19,47.11)	Niger	4.93(2.72,7.93)	Dominica	9.24(5.35,15.06)	Congo	7.51(4.48,11.71)
Finland	36.64(22.13,56.44)	Brazil	13.24(8.25,21.19)	Iraq	9.19(5.34,14.69)	Republic of Moldova	11.25(6.63,17.59)
Botswana	10.11(5.83,16.63)	Barbados	10.22(5.93,16.66)	Mongolia	9.67(5.76,15.59)	Greenland	30.87(18.36,48.01)
Kenya	6.5(3.91,10.43)	Fiji	6.73(3.91,11.09)	Niue	7.12(4.06,11.36)	Malta	28.44(16.88,45.11)
Mali	5.99(3.46,9.61)	Luxembourg	40.79(24.91,63.09)	Republic of Korea	24.51(14.52,39.11)	Cyprus	25.4(15.92,40.28)
Myanmar	6.66(3.87,10.63)	Trinidad and Tobago	12.53(7.28,20.39)	Montenegro	10.47(6.36,16.92)	Cuba	8.82(5.07,14.07)
Qatar	13.85(8.06,23)	Georgia	9.48(5.51,15.4)	Argentina	17.47(10.41,28.46)	Cook Islands	9.24(5.55,15.4)
Haiti	5.99(3.55,9.8)	Kiribati	4.75(2.76,7.58)	North Macedonia	9.35(5.6,14.72)	Sri Lanka	7.75(4.42,12.35)
Samoa	5.95(3.48,9.94)	Honduras	8.06(4.72,13.02)	Senegal	6.56(3.81,10.64)	Eritrea	5.61(3.47,8.91)
Portugal	25.78(15.38,40.94)	Nauru	7.11(4.18,11.19)	Lesotho	6.61(3.91,10.68)	Greece	31.35(19.23,49.05)
Algeria	8.96(5.13,14.42)	Saudi Arabia	11.01(6.42,17.54)	Taiwan (Province of China)	10.39(6.03,16.48)	Monaco	51.2(30.68,79.34)
Cotediv	6.83(3.99,11.15	Colom	10.08(6.03,15.9	Russia	13.28(8.16,21.1	Estonia	13.34(8,21.31)

oire	)	bia	)	n Federation	)		
Puerto Rico	12.9(7.76,21.12)	Lao People's Democratic Republic	6.6(3.91,10.73)	Northern Mariana Islands	8.7(5.17,14.16)	Philippines	6.68(4.07,10.59)
Poland	15.13(9.48,23.37)	Burundi	4.63(2.78,7.41)	Central African Republic	4.67(2.7,7.68)	Grenada	9.88(6.01,15.98)
El Salvador	9.01(5.39,14.44)	United Kingdom	28.35(17.89,44.14)	Namibia	9.1(5.47,14.71)	Gambia	6.01(3.44,9.81)
Somalia	3.22(1.81,5.15)	Guyana	8.99(5.33,14.18)	Chile	18.3(10.73,29.49)	Netherlands	32.55(20.28,48.82)
Benin	6.19(3.6,9.75)	Pakistan	7.1(4.37,11.45)	Croatia	12.88(8.27,19.78)	Malawi	5.22(2.98,8.22)
Jamaica	8.81(5.12,14.41)	Hungary	12.85(7.85,20.93)	Azerbaijan	10.36(6.01,16.91)	Uzbekistan	8.97(5.36,14.26)
Tajikistan	6.95(4.17,11.04)	Kazakhstan	11.9(7.33,19.38)	France	31.74(19.51,50.63)	Morocco	7.9(4.64,12.65)
Costa Rica	10.76(6.39,17.19)	Ethiopia	5.45(3.34,8.86)	Kuwait	14.71(8.77,23.99)	Tonga	5.98(3.41,9.84)
Tuvalu	5.29(3.13,8.81)	Australia	40.87(24.75,64.91)	Israel	20.17(12.04,31.68)	Eswatini	8.72(5.14,13.99)
Belarus	11.73(6.91,18.81)	Turkey	9.49(5.76,15.49)	Zimbabwe	6.3(3.75,10.26)	Switzerland	38.82(25.11,58.6)
Antigua and Barbuda	11.42(6.8,18.27)	New Zealand	32.79(19.06,52.18)	Bolivia (Plurinational State of)	8.22(4.94,13.46)	China	7.42(4.45,11.81)
Armenia	9.19(5.35,14.81)	Lithuania	12.98(7.92,20.19)	Saint Kitts and Nevis	11.86(7.05,19.38)	Equatorial Guinea	10.27(6.01,16.53)

Oman	10.89(6.18,17.56)	Gabon	9.94(5.93,16.14)	Italy	23.92(14.41,37.3)	Sao Tome and Principe	6.72(3.92,10.84)
Guam	10.08(5.65,16.52)	Nigeria	7.23(4.39,11.49)	Andorra	32.5(19.25,50.93)	Mauritius	8.48(4.96,13.32)
Peru	10.01(5.87,16.31)	Canada	25.07(14.85,40.3)	Solomon Islands	4.75(2.69,7.84)	Zambia	6.94(4.03,11.21)
Ukraine	9.71(5.91,15.56)	Belize	8.74(5.25,14.38)	Panama	12.94(7.57,20.81)	Bermuda	14.96(8.63,24.2)
India	7.24(4.45,11.44)	Egypt	8.67(5.16,14.23)	Lebanon	8.82(5.02,14.21)	Tunisia	8.52(4.79,13.92)
Sierra Leone	5.42(3.05,8.74)	Ireland	29.32(17.97,44.77)	United Arab Emirates	12.49(7.15,20.44)	San Marino	28.41(16.64,45.87)
Dominican Republic	10.33(6.04,16.65)	Indonesia	7.05(4.3,11.04)	Ecuador	9.28(5.35,15.08)	Cabo Verde	7.88(4.6,12.22)
Cambodia	5.76(3.41,9.5)	Czechia	14(8.98,21.35)	Ghana	7.49(4.41,12.29)	Guinea	6.05(3.49,9.76)
Austria	30.78(18.99,46.61)	Burkina Faso	5.79(3.35,9.44)	Romania	11.46(6.83,18.75)	Tokelau	6.29(3.76,10.23)

Table S4:The age-standardized DALYs rate of anorexia nervosa across 204 countries and territories in 2021. UI, uncertainty intervals; DALYs, disability-adjusted life years.

Location	ASIR (95%UI) in 2021(per 100,000 population)	Location	ASIR (95%UI) in 2021(per 100,000 population)	Location	ASIR (95%UI) in 2021(per 100,000 population)	Location	ASIR (95%UI) in 2021(per 100,000 population)
Taiwan (Province of China)	17.91(12.46,24.87)	Albania	16.21(11.37,22.42)	Nicaragua	15.86(11.04,22.05)	Democratic Republic of the Congo	10.41(7.39,14.35)
Marshall Islands	11.57(8.13,16.01)	Bulgaria	17.68(12.3,24.56)	Kuwait	23.34(16.37,32.68)	Rwanda	12.24(8.72,16.84)
Cambodia	12.22(8.52,16.66)	Denmark	27.06(18.73,37.9)	Libya	16.18(10.92,22.9)	Kenya	13.06(9.11,17.81)
Sri Lanka	15.11(10.46,2	Czechia	20.1(14.05,27	Costa	19.15(13.17,2	Zambia	13.6(9.52,19.

	1.23)		.85)	Rica	6.51)		18)
Timor-Leste	13.3(9.24,18.53)	Latvia	20.68(14.33,28.88)	Bahrain	20.03(13.69,28.52)	Burundi	10.17(7.14,14.04)
Lao People's Democratic Republic	13.54(9.51,18.71)	Romania	18.76(13.21,26.2)	Saudi Arabia	19.64(13.66,27.53)	Lesotho	13.49(9.35,18.76)
Papua New Guinea	11.6(7.88,16.08)	Austria	27.76(19.57,37.42)	Venezuela (Bolivarian Republic of)	18.25(12.83,25.79)	Somalia	7.61(5.41,10.25)
Maldives	14.9(10.51,20.62)	Germany	28.51(20.24,39.16)	Tunisia	16.8(11.7,23.94)	Malawi	11.05(7.59,15.2)
Democratic People's Republic of Korea	10.58(7.39,14.4)	Ukraine	17.76(12.23,24.62)	Bolivia (Plurinational State of)	16.14(11.26,21.78)	Uganda	12.21(8.46,16.8)
Solomon Islands	10.58(7.34,14.49)	Republic of Moldova	18.01(12.66,25.4)	Guatemala	17.23(12.11,23.78)	Botswana	17.69(12.4,24.52)
Armenia	16.26(11.33,22.59)	Slovakia	18.94(13.18,26.46)	Palestine	14.31(10.13,20.08)	South Africa	16.77(11.71,23.14)
Kiribati	10.62(7.39,14.51)	Republic of Korea	29.08(20.26,39.96)	Iran (Islamic Republic of)	22.75(15.63,32.2)	Namibia	16.6(11.41,22.93)
Vanuatu	11.08(7.67,15.16)	Malta	27.68(19.12,39.41)	Mexico	19.46(13.61,26.67)	Zimbabwe	12.86(9,17.64)
Georgia	16.42(11.68,22.81)	Cyprus	25.34(17.18,36.04)	Brazil	23.21(16.33,32.04)	Burkina Faso	12.08(8.52,16.57)
Kyrgyzstan	14.28(10.1,19.85)	Norway	33.86(23.51,47.21)	Suriname	18.63(12.86,24.8)	Benin	12.7(8.75,17.37)
Philippines	13.59(9.52,18.22)	Iceland	27.61(19.04,39.19)	United Arab Emirates	21.82(14.77,30.36)	Eswatini	16.06(11.24,22.41)
Micronesia (Federated States of)	11.4(7.86,15.67)	Bahamas	21.29(14.95,29.49)	Qatar	23.55(16.1,33.87)	Cameroon	13.25(9.31,18.2)
Indonesia	14.2(9.9,19.18)	Uruguay	24.95(17.4,36.19)	Lebanon	17.15(11.75,23.64)	Cabo Verde	14.81(10.25,20.65)
Thailand	15.88(11.12,2	Israel	17.84(12.13,2	Jordan	16.18(11.13,2	Chad	12.15(8.44,16

	2.18)		4.69)		2.64)		.71)
Azerbaijan	17.56(12.34,24.31)	Spain	40.92(28.45,57.53)	Peru	18.32(12.89,25.13)	Cotedivoire	13.43(9.41,18.44)
Tonga	12.65(8.91,17.51)	Argentina	24.19(16.6,34.1)	Colombia	18.55(12.83,25.67)	Gambia	12.4(8.67,16.97)
China	14.48(10.02,19.76)	France	28.19(19.56,39.57)	El Salvador	17.2(12.12,23.85)	Ghana	14.35(10.1,19.94)
Myanmar	13.48(9.43,18.44)	Guyana	17.27(12,24.01)	Panama	21.47(15.03,30.09)	Guinea	12.48(8.72,17.33)
Samoa	12.47(8.74,17.22)	Switzerland	31.15(22.21,42.42)	Algeria	17.32(12.24,24.32)	Guinea-Bissau	11.86(8.27,16.45)
Viet Nam	13.53(9.43,18.91)	Luxembourg	33.62(23.74,46.87)	Egypt	16.88(11.77,23.15)	Liberia	10.87(7.53,15.02)
Fiji	13.73(9.52,18.99)	Belize	16.94(12.02,23.21)	Bangladesh	14.04(9.66,19.19)	Mauritania	13.63(9.54,18.76)
Malaysia	17.02(11.91,23.76)	Canada	36.23(25.27,50.9)	Syrian Arab Republic	14.79(10.11,20.38)	Mali	12.37(8.64,17.17)
Kazakhstan	19.28(13.68,26.47)	Dominica	17.63(12.33,23.96)	Iraq	17.62(11.98,25.08)	Niger	10.75(7.68,15.35)
Turkmenistan	18.36(12.87,25.58)	Greece	27.87(19.3,38.86)	Angola	14.88(10.34,20.31)	Nigeria	14.44(10.09,19.77)
Mongolia	16.95(12.02,23.2)	Chile	25(17.06,35.33)	Turkey	17.67(12.27,24.99)	Sierra Leone	11.55(7.97,16.07)
North Macedonia	16.65(11.58,23.11)	Netherlands	24.59(17.56,34.52)	Morocco	15.79(10.81,22.25)	Senegal	13.1(9.01,17.89)
Estonia	21.21(14.75,29.48)	Jamaica	17.06(11.9,23.5)	India	14.47(10.17,19.73)	Sao Tome and Principe	13.26(9.13,18.31)
Poland	21.95(15.51,30.33)	Ireland	27.49(18.89,37.92)	Oman	19.9(13.82,28.09)	Togo	11.72(8.09,16.43)
New Zealand	39.21(27.68,55.09)	Antigua and Barbuda	20.2(14.05,28.31)	Congo	14.39(10.19,20.07)	Bermuda	23.76(16.29,33.74)
Lithuania	20.38(14.1,28.38)	Portugal	25.9(17.99,36.98)	Djibouti	13.06(9.01,17.99)	American Samoa	14.57(10.07,20.07)
Bosnia and Herzegovina	16.62(11.59,22.89)	Grenada	18.31(12.79,25.61)	Afghanistan	12.35(8.63,17.16)	Greenland	34.32(23.79,48.25)
Serbia	17.2(12.04,23.86)	Haiti	13.12(9.3,17.88)	Pakistan	14.31(9.91,19.52)	Guam	18.04(12.46,25.25)
Japan	37.27(26.51,5	Finland	36.38(25.48,5	Equatoria	17.32(12.4,23	Cook	17.06(11.96,2

	1.31)		1.43)	l Guinea	.95)	Islands	4.09)
Tajikistan	13.57(9.59,18.8)	Saint Vincent and the Grenadines	17.8(12.32,24.39)	Bhutan	15.66(11.03,22.18)	Nauru	14.26(9.9,19.88)
Croatia	18.9(13.41,25.79)	Italy	22.74(15.85,31.69)	Mozambique	11.39(8.01,15.82)	Monaco	39.76(27.9,56.32)
Andorra	29.81(20.96,41.38)	Sweden	33.59(23.11,49.44)	Ethiopia	11.46(8.05,15.55)	Northern Mariana Islands	16.32(11.04,22.58)
Singapore	33.22(22.66,47.31)	Barbados	18.71(13.08,26.21)	Yemen	12.86(8.88,17.73)	Saint Kitts and Nevis	20.7(14.34,28.54)
Russian Federation	21.63(15.19,30.01)	Cuba	17(11.87,23.61)	Nepal	13.29(9.23,18.38)	Niue	14.16(9.83,19.81)
Slovenia	19.11(13.23,26.26)	Ecuador	17.33(12.01,23.93)	Madagascar	11.66(8.13,15.92)	Puerto Rico	21.88(15.22,30.63)
Belarus	19.4(13.7,27.07)	Dominican Republic	18.95(13.31,25.96)	Central African Republic	10.3(7.25,14.13)	Tokelau	13(9.18,18.01)
Brunei Darussalam	34.52(23.82,47.42)	United States of America	29.35(20.42,39.99)	Seychelles	16.98(11.67,23.33)	San Marino	27.68(18.71,39.68)
Uzbekistan	16.14(11.28,22.35)	Honduras	15.84(11.21,21.8)	Comoros	12.53(8.85,17.23)	Palau	14.35(9.89,19.97)
Hungary	19.53(13.65,26.74)	Paraguay	23.91(16.6,33.23)	Mauritius	16.16(11.23,22.13)	United States Virgin Islands	22.83(15.95,31.66)
Montenegro	17.65(12.65,25.01)	Saint Lucia	18.06(12.27,25.55)	Eritrea	11.64(8.17,15.98)	Tuvalu	11.43(7.93,15.48)
Australia	48.64(34.34,69.67)	Trinidad and Tobago	21.5(14.95,29.78)	Gabon	17.44(12.05,24.49)	South Sudan	11.93(8.21,16.26)
Belgium	28.72(19.68,40.98)	United Kingdom	28.89(20.19,40.19)	United Republic of Tanzania	12.94(9.11,17.72)	Sudan	14.08(9.79,19.5)

Table S5:The ASIR of anorexia nervosa across 204 countries and territories in 2021. UI, uncertainty intervals; ASIR, age-standardized incidence rate.

**Location**	**age-standardized DALYs rate (95%UI) in**	**Location**	**age-standardized DALYs rate (95%UI) in**	**Location**	**age-standardized DALYs rate (95%UI) in**	**Location**	**age-standardized DALYs rate (95%UI) in**

	2021(per 100,000 population)		2021(per 100,000 population)		2021(per 100,000 population)		2021(per 100,000 population)
Thailand	25.32(13.64,43.68)	Uruguay	63.18(35.65,104.51)	Netherlands	54.86(31.88,86.88)	Ghana	25.91(14.69,42.2)
Guinea-Bissau	18.56(10.2,31)	Singapore	78.09(44.4,125.41)	Marshall Islands	15.73(8.8,26.04)	Romania	29.93(16.48,49.32)
Vanuatu	14.83(8.37,24.64)	Germany	60.56(34.79,96.46)	South Sudan	19.28(10.68,32.04)	Turkmenistan	29.96(16.31,50.74)
Slovakia	33.72(18.84,54.01)	Saint Vincent and the Grenadines	42.45(23.54,70.29)	Nicaragua	32.11(18.37,53.12)	Madagascar	18.28(10.37,30.13)
Malaysia	28.99(16.23,48.27)	Sweden	85.12(48.95,138.39)	Kyrgyzstan	19.07(10.73,32.14)	South Africa	34.02(19.21,57.15)
Sudan	28.11(15.09,46.49)	Bulgaria	29.89(15.93,50.57)	Micronesia (Federated States of)	15.44(8.48,25.75)	United States Virgin Islands	68.9(38.62,115.12)
Albania	25.64(14.35,42.39)	Saint Lucia	43.81(23.9,72.86)	Bangladesh	24.07(13.31,38.97)	Togo	18.51(10.22,31.25)
Slovenia	34.52(19.13,57.65)	Bahrain	53.16(29.64,86.09)	Iceland	75.12(44.37,120.54)	Mozambique	16.99(9.89,27.87)
Democratic People's Republic of Korea	11.85(6.45,19.97)	American Samoa	23.98(13.35,40.48)	Chad	19.51(11.42,32.39)	Democratic Republic of the Congo	14.9(8.27,25.38)
Japan	63.45(35.81,105.15)	Comoros	21(11.79,35.76)	Viet Nam	19.37(10.47,33.37)	Suriname	46.3(26.09,76.21)
Angola	27.97(15.35,45.93)	United Republic of Tanzania	21.94(12.34,37.19)	Palau	23.8(13.02,39.28)	Venezuela (Bolivarian Republic of)	42.42(23.3,69.92)
Seychelles	28.69(15.95,47.58)	Cameroon	22.63(12.76,37.38)	Maldives	24.19(13.1,41.01)	Mexico	47.97(27.09,80.2)

Bhutan	30.45(16.26,51.52)	Uganda	19.75(10.84,33.22)	Papua New Guinea	16.09(8.67,26.8)	Paraguay	37.69(20.71,63.7)
Brunei Darussalam	72.86(40.36,119.39)	Nepal	21.95(12.47,37.81)	Latvia	32.31(18.3,53.02)	Bahamas	58.79(32.41,97.61)
Denmark	73.78(42.34,121.47)	Guatemala	36.84(20.38,61.94)	Djibouti	23.81(12.92,40.19)	Iran (Islamic Republic of)	49.17(27.66,80.07)
United States of America	68.86(38.36,114.65)	Mauritania	23.86(12.99,39.85)	Bosnia and Herzegovina	25.72(14.4,42.74)	Yemen	23.74(13.14,38.74)
Finland	75.1(42.58,121.34)	Puerto Rico	62.61(34.98,102.12)	Jordan	35.95(20,59.26)	Belgium	68.55(39.54,110.31)
Norway	85.3(48.98,138)	Niger	15.9(8.78,26.61)	Palestine	29.05(16.57,48.13)	Afghanistan	22.24(12.51,37.06)
United Arab Emirates	57.72(33.86,92.86)	Brazil	41.35(23.22,68.57)	Liberia	15.87(8.75,25.82)	Congo	26.22(14.52,44.54)
Botswana	36.43(20.37,61.22)	Barbados	47.08(26.18,77.66)	Rwanda	19.78(10.98,33.52)	Republic of Moldova	21.08(11.7,35.39)
Kenya	23.23(12.98,39.16)	Luxembourg	91.84(54.18,145.21)	Dominica	41.6(22.88,67.58)	Greenland	84.08(47.5,135.17)
Timor-Leste	18.86(10.42,32.01)	Trinidad and Tobago	60.24(32.98,102.48)	Iraq	41.86(23.41,67.8)	Malta	69.63(40.37,113.85)
Mali	20.02(11.3,33.8)	Georgia	24.82(13.78,41.14)	Mongolia	25.86(14.46,43.71)	Cyprus	67.16(39.06,106.27)
Myanmar	18.8(10.87,31.57)	Kiribati	13.45(7.46,22.63)	Niue	22.95(12.85,37.97)	Cuba	39.04(22.35,65.21)
Qatar	70.34(40.19,113.23)	Honduras	32.02(17.71,54.39)	Republic of Korea	61.05(33.71,100.18)	Cook Islands	30.96(17.35,51.18)
Haiti	24.31(13.65,40.38)	Nauru	22.78(12.49,37.98)	Montenegro	28.57(15.57,45.46)	Serbia	27.85(15.74,46.13)
Samoa	18.17(10.2,30.52)	Saudi Arabia	51.76(29.25,84.15)	Argentina	60.06(34.26,97.48)	Sri Lanka	23(13.27,38.44)

Portugal	64.29(36.61,105.75)	Colombia	44.03(25.05,72.22)	North Macedonia	26.87(15.13,44.28)	Greece	68.54(39.4,111.79)
Algeria	40.7(23.38,67.07)	Lao People's Democratic Republic	19.35(10.67,32.91)	Senegal	22.52(12.47,37.12)	Monaco	142.15(82.83,230.51)
Cotedivoire	23.8(12.88,39.48)	Burundi	14.4(7.86,23.87)	Lesotho	22.08(12.85,36.95)	Eritrea	18.76(10.2,31.76)
Poland	33.93(18.9,57.21)	United Kingdom	74.03(41.85,120.25)	Taiwan (Province of China)	31.85(17.56,52.72)	Estonia	33.63(18.52,56.82)
El Salvador	37.3(21.3,61.51)	Guyana	40.12(22.23,65.55)	Russian Federation	32.38(18.12,54.33)	Philippines	20.39(11.28,33.69)
Somalia	9.13(4.98,15.21)	Pakistan	26.45(14.85,44.09)	Northern Mariana Islands	29.59(16.7,49.58)	Grenada	45.06(24.91,73.85)
Benin	20.88(12.02,34.39)	Hungary	34.77(19.28,57.18)	Italy	100.43(60.3,159.7)	Gambia	20.08(11.23,33.71)
Jamaica	39.18(21.81,65.02)	Kazakhstan	32.02(17.76,52.97)	Central African Republic	14.6(8.36,24.36)	Malawi	16.61(9.01,27.91)
Tajikistan	17.52(9.89,28.73)	Syrian Arab Republic	30.07(17.1,49.31)	Namibia	32.63(17.78,54.12)	Uzbekistan	23.45(13.05,38.95)
Costa Rica	46.07(25.14,77.83)	Ethiopia	19.36(10.68,32.32)	Chile	64.23(37.32,105.14)	Morocco	34.67(19.77,57.12)
Tuvalu	15.98(8.72,26.5)	Australia	185.04(116.93,281.98)	Croatia	31.42(17.71,51.84)	Tonga	18.48(10.04,30.32)
Belarus	29.15(16.13,48.6)	Turkey	52.91(29.67,86.99)	Azerbaijan	28.25(15.73,47.9)	Eswatini	30.38(16.8,50.35)

Antigua and Barbuda	53.98(30.26,89.74)	New Zealand	99.89(57.18,166.5)	France	76.76(45.37,125.18)	Switzerland	64.68(37.39,102.65)
Armenia	24.26(13.92,39.65)	Lithuania	33.8(18.71,56.54)	Kuwait	64.19(35.76,104.29)	China	22.18(12.29,36.92)
Oman	51.97(29.27,83.62)	Libya	35.63(20.07,56.58)	Israel	62.3(34.91,100.67)	Sao Tome and Principe	22.78(12.79,38.37)
Guam	35.51(19.74,59.36)	Gabon	36.45(20.32,60.07)	Zimbabwe	20.28(10.8,34.08)	Mauritius	27(15.11,46.9)
Peru	59.25(32.56,100.85)	Nigeria	26.86(15.07,45.5)	Saint Kitts and Nevis	56.22(30.95,91.3)	Equatorial Guinea	40.1(21.84,65.6)
Ukraine	24.16(13.65,40.02)	Canada	70.9(41.04,114.21)	Fiji	21.17(11.59,35.38)	Zambia	23.5(13.23,39.24)
India	27.7(15.68,46.85)	Belize	38.94(21.46,65.04)	Andorra	84.44(47.9,134.82)	Bermuda	73.73(41.38,121.5)
Spain	90.69(51.47,146.47)	Egypt	38.89(21.39,63.18)	Bolivia (Plurinational State of)	49.06(27.56,82.56)	Tunisia	38.57(22.04,63.29)
Sierra Leone	17.71(10.07,29.87)	Indonesia	22.2(12.3,37.73)	Solomon Islands	13.65(7.55,22.77)	San Marino	79.14(45.77,131.93)
Dominican Republic	48.07(26.46,79.43)	Ireland	79.1(45.78,128.28)	Panama	51.05(27.94,84.17)	Cabo Verde	28.01(15.94,46.65)
Cambodia	16.29(8.81,27.44)	Czechia	34.89(19.62,57.75)	Lebanon	40(21.99,64.85)	Guinea	20.21(11.23,33.76)
Austria	84.81(49.23,136.75)	Burkina Faso	19.09(10.41,31.43)	Ecuador	56.4(31.5,94.48)	Tokelau	19.64(10.4,33.11)

Table S6:The age-standardized DALYs rate of bulimia nervosa across 204 countries and territories in 2021. UI, uncertainty intervals; DALYs, disability-adjusted life years.

Location	ASIR (95%UI) in 2021(per 100,000 population)	Location	ASIR (95%UI) in 2021(per 100,000 population)	Location	ASIR (95%UI) in 2021(per 100,000 population)	Location	ASIR (95%UI) in 2021(per 100,000 population)

Indonesia	95.81(59.37,145.79)	Austria	137.54(89.66,195.19)	Peru	123.93(78.47,182.48)	United Republic of Tanzania	78.34(49.72,115.89)
Malaysia	93.7(59.39,141.01)	Andorra	143.06(92.56,205.28)	Suriname	115.69(71.81,172.5)	Uganda	74.95(47.59,111.63)
Myanmar	73.36(46.69,109.56)	Belgium	129.28(83.39,182.84)	Colombia	118.01(75,175.21)	Central African Republic	64.14(40.37,96.7)
Thailand	85.68(53.39,128.68)	Germany	122.37(79.54,172.77)	El Salvador	105.5(66,159.88)	Somalia	52.18(32.6,80.57)
Viet Nam	76.06(48.61,115.09)	Cyprus	125.96(82.7,176.31)	Costa Rica	119.27(74.07,176.25)	Democratic Republic of the Congo	66.2(41.54,99.08)
Samoa	73.76(45.97,107.95)	Denmark	142.71(94.02,202.67)	Honduras	97.86(61.05,145.18)	Gabon	101.09(64.95,150.53)
Tonga	73.22(45.85,108.4)	France	132.57(87.28,184.76)	Mexico	142.72(87.81,209.81)	Equatorial Guinea	114.89(71.8,172.23)
Kazakhstan	101.28(64.92,152.83)	Finland	134.32(87.96,187.92)	Egypt	116.9(74.66,171.54)	Zambia	82.05(51.65,123.25)
China	101.51(62.58,155.5)	Russian Federation	120.52(74.87,180.75)	Madagascar	72.12(45.9,107.35)	Malawi	67.85(43.78,101.44)
Albania	95.07(61.44,141.48)	Ukraine	104.15(65.9,154.81)	Nicaragua	99.35(61.33,148.15)	Tokelau	77.52(49.1,116.69)
Democratic People's Republic of Korea	60.47(37.63,92.01)	Lithuania	103.73(66.21,152.23)	Venezuela (Bolivarian Republic of)	111.92(70.72,162.46)	Benin	76.72(48.43,114.22)
Cambodia	69.36(44,106.78)	Greece	127.67(82.66,178.51)	Panama	126.79(78.89,186.15)	Ghana	86.13(54.73,127.37)
Bulgaria	101.2(65.58,148.28)	Iceland	133.62(86.99,193.9)	Ecuador	120.49(74.46,173.93)	Cabo Verde	91.56(57.29,136.07)
Taiwan (Province of China)	99.78(62.05,148.53)	Ireland	139.32(90.14,198.35)	Iraq	122.3(78.12,174.39)	Mauritania	81.99(51.85,122.95)
Fiji	79.72(49.89,119.62)	Portugal	123.92(80.48,173.78)	Kuwait	152.17(99.24,216.32)	Liberia	68.31(42.78,103.49)
Marshall Islands	68.41(43.08,104.72)	Israel	105.67(69.58,148.84)	Seychelles	94.21(61.43,140.88)	Guinea-Bissau	72.16(45.69,107.11)

Lao People's Democratic Republic	75.75(48.06,112.69)	Netherlands	90.29(59.71,127.78)	Bolivia (Plurinational State of)	113.06(71.2,167.64)	Chad	74.06(47.32,111.33)
Brunei Darussalam	154.23(96.66,225.63)	Japan	169.61(105.45,249.04)	Bahrain	149.96(96.13,213.75)	Gambia	75.46(47.63,114.34)
Philippines	91.33(56.56,139.96)	Italy	133.18(86.72,188.38)	Iran (Islamic Republic of)	158.77(101.43,228.26)	Burkina Faso	72.43(45.06,110.56)
Maldives	90.51(57.79,136.26)	Luxembourg	148.8(96.31,211.68)	Palestine	100.39(64.6,144.96)	Cameroon	80.86(51.1,120.44)
Micronesia (Federated States of)	67.95(42.18,101.91)	Malta	128.2(83.79,183.34)	Algeria	118.85(77.12,170.84)	Niger	67.17(41.83,99.74)
Timor-Leste	74.27(46.14,113.02)	Norway	170.53(110.03,244.67)	Turkey	130.42(84.43,188.7)	Guinea	74.93(47.14,111.96)
Sri Lanka	82.09(51.29,121.68)	Singapore	158.08(101.2,227.4)	Jordan	114.29(74.32,167.47)	Nigeria	102.24(63.01,155.66)
Papua New Guinea	69.86(43.28,105.54)	United Kingdom	146.55(95,209.32)	Lebanon	119.72(77.58,174.16)	Botswana	103.35(66.1,153.76)
Kiribati	62.33(39.03,95.03)	Spain	187.86(122.39,269.07)	Libya	111.33(72.45,160.15)	Lesotho	80.41(49.27,120.48)
Bosnia and Herzegovina	93.83(59.9,137.24)	Sweden	347.13(214.25,527.83)	Yemen	90.33(57.33,132.31)	Eswatini	94.09(60.48,141.58)
Mongolia	90.17(57.78,131.77)	Republic of Korea	140.46(89.45,204.03)	Afghanistan	88.62(56.23,128.92)	Senegal	81.52(51.97,122.44)
Vanuatu	66.28(41.67,101.96)	Australia	289.42(193.08,407.76)	Burundi	63.56(40.88,94.26)	Zimbabwe	75.57(48.37,112.78)
Solomon Islands	63.54(40.02,95.16)	Switzerland	125.65(81.3,179.17)	Morocco	110.13(71.29,160.43)	Mali	75.56(47.18,115.1)
Armenia	88.42(56.58,132.9)	Canada	145.28(91.78,210.69)	Oman	145.18(95.31,209.62)	South Africa	119.8(73.66,181.82)
Azerbaijan	94.93(60.66,137.87)	Bahamas	129.76(82.05,191.84)	Qatar	180.37(115.71,259.79)	Togo	72.89(45.63,110.73)
Turkmenistan	99.54(62.88,149.53)	Argentina	134.76(87.24,191.32)	India	107.66(66.55,163.19)	Namibia	96.95(61.67,143.38)
Estonia	104.25(65.49,156.85)	Chile	141.4(90.03,201.95)	Cotedivoire	83.89(52.6,128.26)	San Marino	137.09(88.97,193.71)
Kyrgyzsta	77.27(49.15,1	Uruguay	138.92(86.89,	Paraguay	106.65(65.72,	Cook	93.61(58.11,1

n	15.38)		197.97)		158.75)	Islands	38.49)
Georgia	89.62(57.73,136.67)	Belize	105.37(65.02,155.14)	Tunisia	115.76(74.32,168.06)	Sao Tome and Principe	81.14(51.19,122.05)
Tajikistan	74.36(47.48,111.74)	Antigua and Barbuda	124.26(78.82,182.41)	Bhutan	95.54(60.8,142.1)	Sierra Leone	70.74(43.85,105.9)
Hungary	108.07(69.41,159.59)	Dominican Republic	118.18(74.39,176.46)	Nepal	78.6(48.41,118.38)	Bermuda	145.71(91.56,209.05)
New Zealand	247.54(153.5,365.64)	Guyana	107.64(66.74,162.16)	Pakistan	105.23(65.63,159.25)	American Samoa	84.76(53.59,125.5)
Uzbekistan	86.79(55.57,130.62)	Jamaica	105.91(66.51,155.43)	Brazil	131.6(80.44,196.34)	Puerto Rico	133.86(83.76,199.18)
Czechia	110.05(70.14,162.55)	Barbados	115.43(72.18,167.89)	Syrian Arab Republic	97.08(62.87,140.27)	Tuvalu	70.32(44.28,105.23)
North Macedonia	96.07(62.24,142.88)	Grenada	115.14(72.02,170.45)	Bangladesh	82.25(51.77,123.58)	South Sudan	74.06(46.57,111.45)
Montenegro	99.54(63.35,148.77)	United States of America	181.79(113.46,269.78)	Djibouti	85.67(54.12,129.26)	Guam	104.7(65.36,156.19)
Poland	127.72(80.95,191.27)	Cuba	107.11(67.89,159.33)	Comoros	78.02(49.17,116.2)	United States Virgin Islands	140.83(88.13,208.94)
Croatia	104.34(65.81,153.96)	Saudi Arabia	144.49(95.39,207.89)	Mauritius	89.42(55.76,132.79)	Northern Mariana Islands	95.08(60.21,142.09)
Romania	103.61(66.37,151.64)	Dominica	110.5(68.52,165.09)	Angola	89.43(56.79,132.61)	Saint Kitts and Nevis	127.5(78.35,192.7)
Slovakia	107.93(67.98,156.75)	Haiti	83.38(51.97,124.06)	Congo	86.47(54.42,130.25)	Monaco	190.74(126.52,265.51)
Slovenia	109.82(70.81,162.95)	United Arab Emirates	154.08(101.94,220.82)	Eritrea	74.19(46.26,111.87)	Niue	83.62(52.89,125.27)
Serbia	98.87(62.81,147.62)	Saint Lucia	113.02(69.84,164.55)	Ethiopia	87.74(54.37,134.72)	Sudan	98.1(64.13,142.69)
Belarus	96.72(60.96,142.66)	Saint Vincent and the Grenadin	110.94(68.19,165.48)	Kenya	96.27(59.2,146.44)	Nauru	82.75(51.14,123.38)

		es					
Latvia	102.29(64.19, 151.56)	Trinidad and Tobago	132.84(84.08, 195.51)	Mozambique	68.75(43.51,101.66)	Palau	88.53(55.61,134.49)
Republic of Moldova	81.9(53.39,122.29)	Guatemala	105.33(65.67, 156.23)	Rwanda	74.86(47.14,111.83)	Greenland	161.31(103.62,233.09)

Table S7:The ASIR of bulimia nervosa across 204 countries and territories in 2021. UI, uncertainty intervals; ASIR, age-standardized incidence rate.

Age	Age-standardized DALYs rate(95%CI)		ASIR(95%CI)	
	Males	Females	Males	Females
5-9 years	1.24(0.52,2.5)	1.96(0.78,4.02)	9.6(4.9,16.01)	10.73(5.32,20.08)
10-14 years	19.02(10.51,34.6)	31.19(18.25,53.75)	232.61(122.89,406.97)	194.8(117.13,312.98)
15-19 years	64.99(34.03,117.73)	113.75(65.34,192.28)	594.3(291.23,1086.18)	445.93(252.76,745.78)
20-24 years	83.1(39.29,149.82)	160.85(85,260.84)	480.99(248.69,860.86)	356.08(205.97,573.2)
25-29 years	80.68(42.85,136.6)	149.62(83.61,242.58)	280.32(176.25,418.67)	178.1(120.39,253.51)
30-34 years	75.4(39.96,129.41)	117.33(68.98,187.07)	135.06(79.45,209.94)	68.01(41.57,105.15)
35-39 years	57.08(30.2,93.3)	85.09(49.73,134.95)	49.45(30.27,75.46)	30.2(19.87,43.53)
40-44 years	28.35(14.99,45.6)	52.38(30.26,81.15)	4.45(2.84,6.57)	8.3(5.24,12.15)
45-49 years	11.24(5.87,19.81)	27.75(15.84,43.85)	1.49(0.95,2.21)	2.77(1.75,4.05)

Table S8:The age-standardized DALYs rate (B) and ASIR (C) per 100,000 people of eating disorders by age and sex in 2021. DALYs, disability-adjusted life years; ASIR; age-standardized incidence rate.
